# DNA-PK inhibition enhances gene editing efficiency in HSPCs for CRISPR-based treatment of X-linked hyper IgM syndrome

**DOI:** 10.1016/j.omtm.2024.101297

**Published:** 2024-07-27

**Authors:** Cole M. Pugliano, Mason Berger, Roslyn M. Ray, Kai Sapkos, Betty Wu, Aidan Laird, Yidian Ye, Daniel Thomson, M. Quinn DeGottardi, Iram F. Khan, Kristina Tatiossian, Brodie A. Miles, Florian Aeschimann, Jerome Pasquier, Mihee M. Kim, David J. Rawlings

**Affiliations:** 1 Center for Immunity and Immunotherapies and the Program for Cell and Gene Therapy, Seattle Children’s Research Institute, Seattle, WA 98101, USA; 2 Gene Therapy Research, CSL Behring, Pasadena, CA 91101, USA; 3 CSL Behring, Research Bern, 3000 Bern, Switzerland; 4 Swiss Institute for Translational Medicine, Sitem-insel, 3010 Bern, Switzerland; 5 Department of Pediatrics, University of Washington, Seattle, WA 98105, USA; 6 Department of Immunology, University of Washington, Seattle, WA 98109, USA

**Keywords:** CD40L, HPSCs, DNA-PKcs inhibitor, AZD, HDR, CRISPR, AAV6, T cell

## Abstract

Targeted gene editing to restore CD40L expression via homology-directed repair (HDR) in CD34^+^ hematopoietic stem and progenitor cells (HSPCs) represents a potential long-term therapy for X-linked hyper IgM syndrome. However, clinical translation of HSPC editing is limited by inefficient long-term engraftment of HDR-edited HSPCs. Here, we ameliorate this issue by employing a small-molecule inhibitor of DNA-PKcs, AZD7648, to bias DNA repair mechanisms to facilitate HDR upon CRISPR SpCas9-based gene editing. Using AZD7648 treatment and a clinically relevant HSPC source, mobilized peripheral blood CD34^+^ cells, we achieve ∼60% HDR efficiency at the *CD40LG* locus and enhanced engraftment of HDR-edited HSPCs in primary and secondary xenotransplants. Specifically, we observed a 1.6-fold increase of HDR-edited long-term HSPCs in primary transplant recipients without disturbing chimerism levels or differentiation capacity. As CD40L is primarily expressed in T cells, we demonstrate T cell differentiation from HDR-edited HSPCs *in vivo* and in artificial thymic organoid cultures, and endogenously regulated CD40L expression following activation of *in-vivo*-derived CD4^+^ T cells. Our combined findings demonstrate HDR editing at the *CD40LG* locus at potentially clinically beneficial levels. More broadly, these data support using DNA-PKcs inhibition with AZD7648 as a simple and efficacious addition to HSPC editing platforms.

## Graphical abstract

**Figure:**
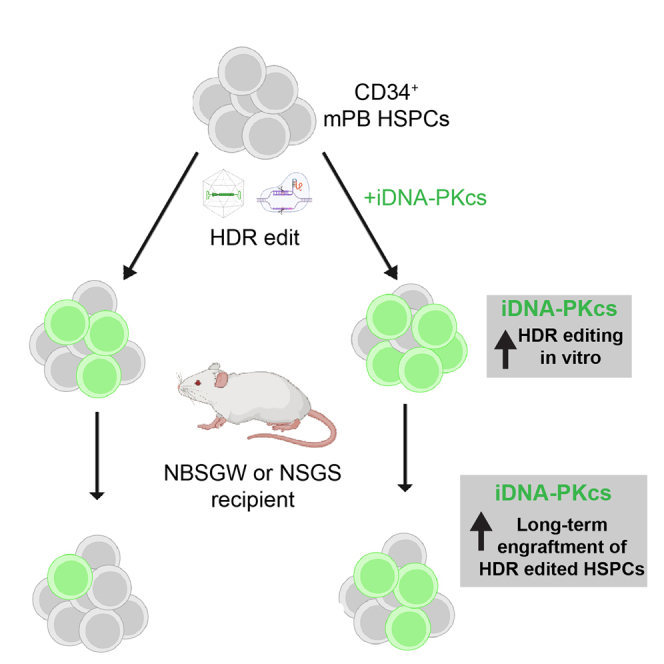

## Teaser

Pugliano and colleagues demonstrate enhanced HDR editing and engraftment of edited hematopoietic stem cells via addition of DNA-PK inhibition during *ex vivo* editing. They use this approach to improve efficiency of HDR-based editing therapy for X-linked hyper IgM syndrome, an immune disorder caused by mutations in the *CD40LG* gene.

## Introduction

X-linked hyper IgM syndrome (XHIM) is a primary immune disorder caused by inactivating mutations in the *CD40LG* gene, which encodes CD40 ligand (CD40L). Mainly expressed on the surface of activated CD4 T cells, CD40L triggers CD40-dependent activation of B cells and other antigen-presenting cells.^1^^,^^2^ In CD40-dependent B cell activation, the CD40:CD40L interaction promotes germinal center formation, class switch recombination, somatic hypermutation, and formation of plasma and memory B cells.^3^ In XHIM patients, CD40L deficiency results in failure to generate protective class-switched, pathogen-specific, high-titer antibodies leading to high susceptibility to recurrent and opportunistic infections^4^ and reduced long-term survival.^5^ Allogeneic hematopoietic stem cell (HSC) transplant offers long-term therapeutic benefit, but risks graft rejection, infection, and requires matched donor availability.^5^

As XHIM is a monogenic disorder, previous studies have aimed to identify approaches to restore expression of wild-type (WT) CD40L protein in XHIM T cells or hematopoietic stem and progenitor cells (HSPCs). CD40L expression achieved via retroviral delivery of a CD40L cDNA expression cassette in CD40L^−/−^ mice resulted in most animals developing lymphoproliferative disorders, highlighting the importance of maintaining endogenous transcriptional control of CD40L expression.^6^ XHIM-causing mutations have been identified throughout the *CD40L* gene and lack a dominant mutational hotspot.^7^ Therefore, precise correction by homology-directed repair (HDR)-based editing with short oligonucleotide donor cassettes^8^ or, alternatively, via base- or prime-editing approaches^9^^,^^10^ would require development of multiple patient-specific designs, not feasible for broad clinical application. In contrast, HDR-based gene editing to insert CD40L cDNA downstream of the *CD40LG* promoter elements allows for restoration of endogenous functional gene expression and has been described in both human T cells and HSPCs.^11^^,^^12^^,^^13^ In female carriers of XHIM, skewed random X chromosome inactivation suggests as little as 10% HDR in HSPCs might be sufficient for therapeutic benefit, assuming efficient engraftment and transgene expression and function.^14^

Due to the simultaneous self-renewal and multilineage differentiation capacity of long-term hematopoietic stem cells (LT-HSCs), efficient HDR editing in LT-HSCs could provide long-term therapeutic benefit in XHIM. However, while efficient *ex vivo* HDR editing in CD34^+^ HSPCs has been reported for multiple blood and immune disorders, xenotransplantation studies consistently demonstrate inefficient long-term engraftment of HDR-edited cells.^15^^,^^16^^,^^17^^,^^18^^,^^19^^,^^20^ Furthermore, in comparison with fetal or neonatal HSPC sources (fetal liver or cord blood), the engraftment deficit of HDR-edited HPSCs is most evident using clinically relevant, mobilized peripheral blood (mPB) HSPCs.^21^

HDR in LT-HSCs is directly related to cell-cycle-dependent DNA repair mechanisms. In the context of genome editing and delivery of homologous DNA donor template, repair of the nuclease-mediated double-stranded DNA break (DSB) occurs via two competing pathways, non-homologous end joining (NHEJ) vs. HDR. NHEJ is rapid and cell-cycle independent, whereas HDR is a slower repair pathway that occurs predominantly in S/G2.^22^^,^^23^ Consistent with these observations, engraftment enriched, CD34^+^CD38^–^ HSPCs are quiescent (G0) and biased toward NHEJ repair.^24^

NHEJ repair is mediated by Ku70/Ku80 recruitment to the DSB, followed by catalytic subunit of DNA-dependent protein kinase (DNA-PKcs) to facilitate DNA end processing and ligation.^25^ DNA-PKcs inhibitors can enhance HDR by inhibiting NHEJ.^26^^,^^27^ Notably, a recently identified DNA-PKcs inhibitor with superior selectivity, AZD7648,^28^ has been shown to enhance HDR efficiency *in vitro* in cell lines, primary cells, and umbilical cord blood-derived HSPCs.^29^^,^^30^^,^^31^

We previously described efficient rAAV6-based HDR editing in primary T cells as a potential therapeutic strategy for XHIM.^11^ Importantly, the expression kinetics of CD40L in gene-edited T cells mirrored that of the endogenous protein in unedited T cells and rescued CD40L expression and function in XHIM T cells. While promising as a T cell therapy, translation of this approach to HSPCs would provide potential curative treatment for XHIM. Here, we report efficient and sustained HDR editing at the *CD40LG* locus using clinically relevant mPB HSPCs. The addition of AZD7648 to our editing protocols resulted in significantly enhanced HDR and superior long-term engraftment of HDR-edited HSPCs. In addition, *CD40LG*-edited HSPCs differentiated into T cells *in vivo* with lineage-restricted and endogenously regulated, CD40L expression.

## Results

### Establishment and optimization of SpCas9-based editing at *CD40LG* in CD34^+^ HSPCs

We previously reported efficient editing of the *CD40LG* locus in human primary T cells with rAAV6 donor template and TALEN nuclease.^11^ To establish and compare this approach with a CRISPR-SpCas9 editing strategy, we designed an sgRNA targeting the 5′ UTR of *CD40LG* and an rAAV6 donor template to deliver a fluorescent reporter cassette. The rAAV donor template utilized a constitutively active MND promoter driving GFP expression followed by WPRE and synthetic poly-adenylation signal elements flanked by 1 kB homology arms (Figure S1A).

As the SpCas9 and TALEN target sites were separated by only 15 bp, we initially employed an identical MND.GFP AAV donor vector for both nucleases, designed to avoid recutting of the repaired DNA locus by either nuclease. TALEN and SpCas9 RNP (SpCas9 protein + *CD40LG* sgRNA) were tested, in parallel, in mPB CD34^+^ HSPCs following the timeline outlined in Figure S1B. At matched rAAV6 multiplicity of infection (MOI), SpCas9 outperformed TALEN in preserving HSPC viability while enabling superior HDR efficiency (Figures S1C and S1D). HDR rates quantified by FACS-matched genomic HDR rates quantified by in-out ddPCR (Figure S2A). After identifying SpCas9 as the preferable nuclease, we generated an optimized MND.GFP AAV donor without previous mutations designed to eliminate TALEN binding, thus increasing the homologous stretch within the 5′ homology arm. This donor resulted in enhanced HDR efficiencies and was used in all subsequent studies.

Next, we assessed the incorporation of the small-molecule drugs, SR1 and UM171, previously reported to support survival and possible *ex vivo* expansion of phenotypically primitive HSPCs^32^^,^^33^ into our HSPC culturing protocol. Culturing in the presence of SR1 and UM171 trended toward higher cell viability at 48 h post-editing in mock-treated, AAV-treated, and AAV+RNP-treated conditions relative to the matched condition cultured without SR1 and UM171 (Figure S3A). Based upon flow cytometry analysis, roughly twice the proportion of phenotypically primitive, HSC-enriched, CD34^+^CD38^−^CD90^+^CD133^+^ cells were present in culture 24 h after editing with SR1 and UM171 treatment with similar fold-expansion independent of treatment with AAV or AAV+RNP (Figure S3B). Similar HDR efficiencies were observed at 1E3 or 2.5E3 MOI of AAV with SR1 and UM171 treatment (Figure S3C).

Following the initial testing of the candidate *CD40LG* sgRNA for cutting efficiency, we next sought to characterize its off-target profile in CD34^+^ mobilized mPB HSPCs using CAST-seq.^34^ CAST-seq detects and quantifies chromosomal rearrangements between off- and on-target sites, resulting in off-target site identification with high sensitivity. In addition, the assay detects chromosomal aberrations mediated by on-target activity only, such as homology-mediated translocations (HMTs), large deletions, and inversions. On-target cleavage was highly efficient (Figure S4A) and a SpCas9-typical pattern of large deletions and inversions at the *CD40LG* target site was detected only in the edited samples (Figures S4B and S4C), providing evidence that the editing and CAST-seq protocol worked as expected. While no HMTs were observed for the *CD40LG* sgRNA, only four low-probability off-target sites were detected by CAST-seq, all with few hits and low gRNA target site alignment scores (Figures S4D and S4E). Notably, the detection of each off-target site was significant in one out of four replicates only. Our findings suggest that the *CD40LG* sgRNA utilized for our studies is highly specific and is likely a suitable sgRNA for therapeutic applications.

Together, these results demonstrate an efficient, specific CRISPR-SpCas9-based HDR editing strategy at the *CD40LG* locus in mPB CD34^+^ HSPCs and optimization of HSPC culturing parameters. However, we anticipated that further development to maximize HDR efficiency within LT-HSC would be required to establish a clinically translatable therapy for XHIM syndrome.

### DNA-PK inhibitor, AZD7648, promotes HDR in CD34^+^ HSPCs with minimal toxicity

**Figure 1:**
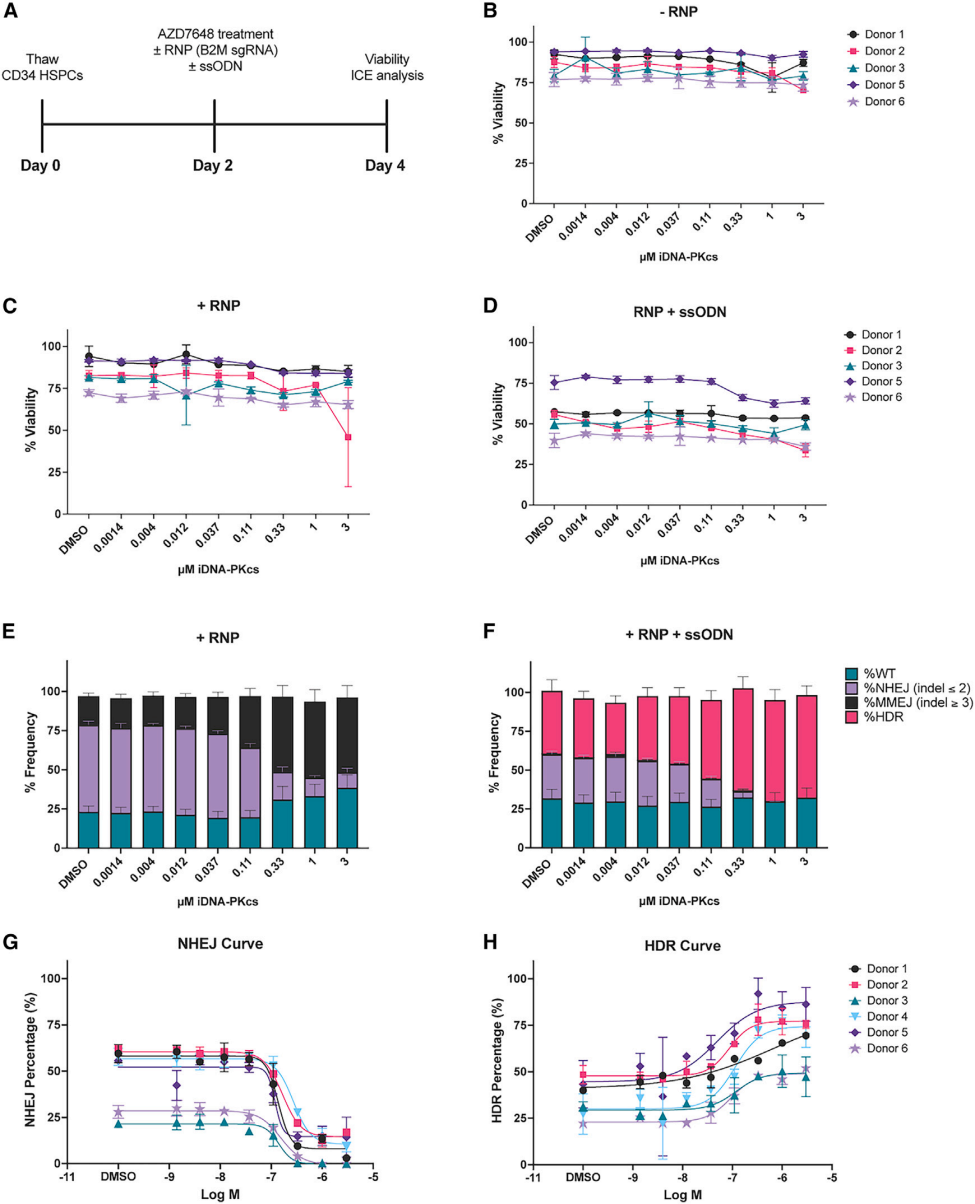
DNA-PK inhibitor AZD7648 inhibits NHEJ and promotes HDR in CD34^+^ HSPCs with minimal toxicity (A) Study design to characterize viability and editing outcomes in CD34^+^ HSPCs. After thaw, CD34^+^ cells were edited with SpCas9 complexed with an sgRNA targeting *B2M*, with and without an ssODN donor template and inhibition of DNA-PKcs with AZD7648. Cells were assessed for viability and editing outcomes 48 h post-editing. (B–D) Viability measured by FACS (SSC-A vs. AAD-7) 48 h after treatment with (B) AZD7648, (C) AZD7648 and RNP, or (D) AZD7648, RNP, and ssODN. (E and F) DNA repair outcomes 48 h after treatment with (E) AZD7648 and RNP or (F) AZD7648 and RNP+ssODN. Indel profiles were evaluated using ICE analysis. NHEJ profiles were counted as ≤ ±2 bp indels, while MMEJ profiles were scored as ≥ ±3 bp indels. Histograms are represented as percent, WT, NHEJ, MMEJ, or HDR of the total editing outcome for each treatment group per donor tested. (G and H) NHEJ frequency (left) and HDR frequency (right) 48 h after treatment with AZD7648 and RNP ± ssODN. A four-parameter logistic (non-linear regression model was used to determine the IC_50_ (NHEJ) and EC_50_ (HDR). (B–H) Data are representative of *n* = 5–6 CD34^+^ donors, with each donor performed as an independent experiment with two to four replicates per experiment. Bars represent mean ± SEM.

Based upon reported specificity and selectivity of the DNA-PKcs inhibitor (iDNA-PKcs), AZD7648,^28^ we initiated experiments to determine whether AZD7648 could modulate HDR in mPB CD34^+^ HSPCs. As a rapid initial assay to evaluate DNA repair outcomes,^35^ we electroporated cells with RNPs targeting the *B2M* gene with or without a small single-stranded oligonucleotide (ssODN) donor template, and subsequently dosed with increasing concentrations of AZD7648. Based on the variability of DNA repair across human subjects,^36^^,^^37^ cells from multiple donors were evaluated for HDR frequency. Viability and editing outcomes were assessed 48 h post-editing (Figure 1A). iDNA-PKcs alone or in combination with RNP ± ssODN, did not dramatically impact cell viability (Figures 1B–1D) at the concentrations tested, and donor template itself had the greatest impact on cell viability (Figure 1D).

To evaluate the efficacy of iDNA-PKcs on the NHEJ pathway, and its consequential impact on HDR, we measured indel frequency and HDR outcomes using inference of CRISPR edits (ICE) analysis.^38^ We observed a dose-dependent decrease in NHEJ frequency (≤2 bp indel) that corresponded with an increase in MMEJ (≥3 bp indel) frequency^35^ with increasing concentrations of iDNA-PKcs (Figure 1E). For groups that included the donor template (Figure 1F; RNP+ssODN), the MMEJ increase observed in Figure 1E was largely replaced with HDR. Similarly, the loss of NHEJ with increasing concentrations of iDNA-Pkcs corresponded with an increase in HDR (Figure 1F). Maximal improvements to HDR-editing profiles were observed with 0.33, 1, and 3 μM iDNA-PKcs.

Next, we analyzed data across donors to obtain the relative IC_50_ and EC_50_ values of AZD7648 on editing outcomes. Inhibition of DNA-PKcs was robust (Figure 1G), with an average IC_50_ of 0.165 μM (SD ± 0.05 μM, Table S1). Similarly, AZD7468 increased HDR across donors (Figure 1H), with an average EC_50_ of 0.33 μM (SD ± 0.3 μM, Table S2). The HDR-EC_50_ was more variable compared with the NHEJ-IC_50_ with the variability of starting HDR and HDR_Max_ frequencies highlighting the importance of understanding HDR constraints at the donor level.

### iDNA-PKcs enables enhanced HDR editing at the *CD40LG* locus in CD34^+^ HSPCs

**Figure 2:**
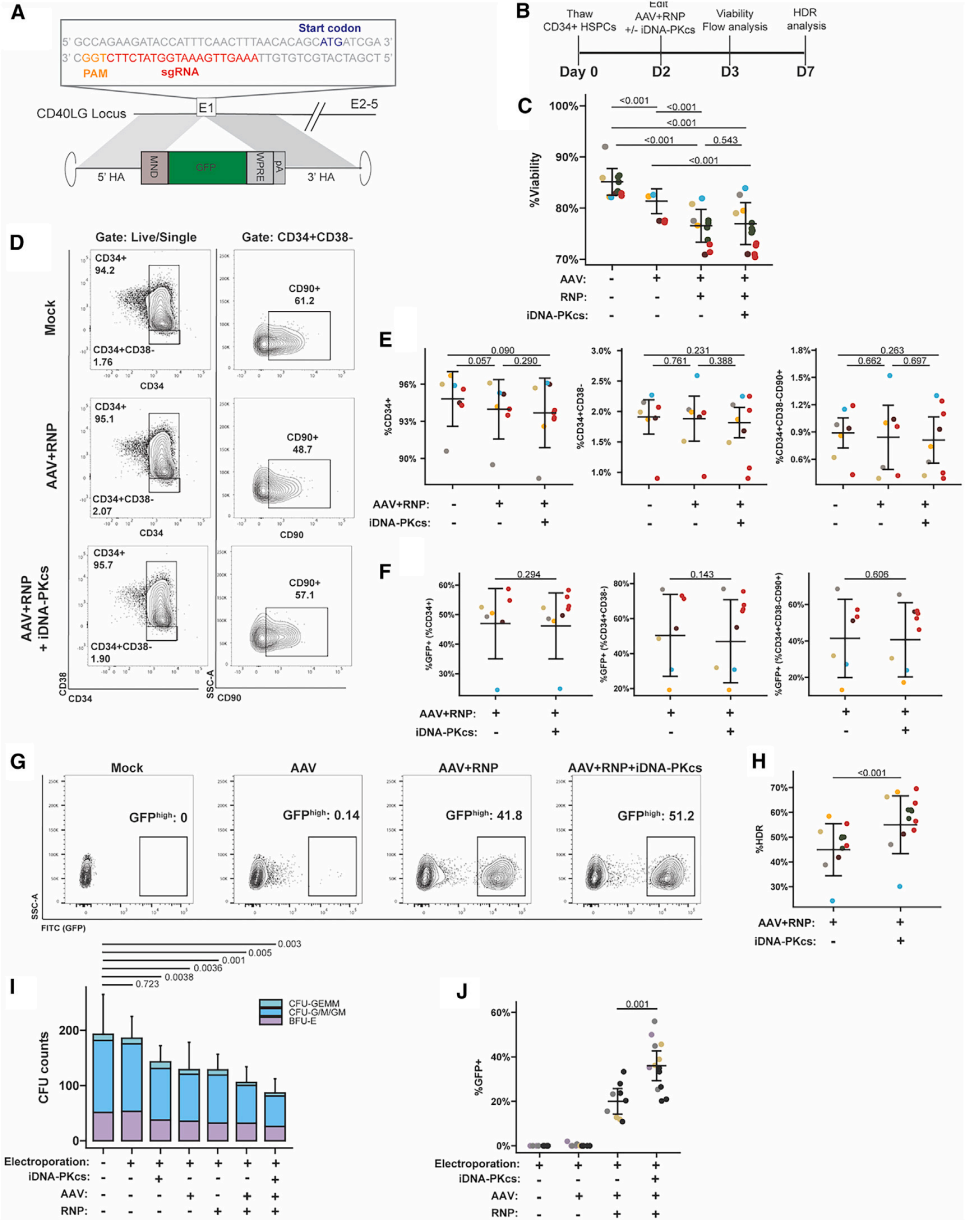
Inhibition of DNA-PKcs enhances AAV-based HDR editing efficiency in CD34^+^ HSPCs (A) Schematic for CRISPR-Cas9 targeting of *CD40LG* exon 1 (E1). Above E1, the sgRNA sequence and location in relation to the start codon and PAM sequence is highlighted. Below E1, schematic of MND.GFP HDR donor template containing 1 kB homology arms flanking a constitutive MND promoter-driven GFP cassette followed by WPRE and synthetic polyadenylation (pA) regulatory elements. (B) Study design to characterize HSPC phenotype and HDR efficiency with rAAV6+RNP-based HDR editing and inhibition of DNA-PKcs. (C) Viability of HSPCs at 24 h after the indicated treatments, *n* = 2–4 CD34^+^ donors from 2 to 3 independent experiments. (D) Representative flow cytometry analysis of HSPC phenotype 24 h after the indicated treatments. (E) Proportion of CD34^+^ (left), CD34^+^CD38^–^ (middle), and CD34^+^CD38^−^CD90^+^ (right) HSPCs as percentage of total cells 24 h after editing, editing in combination with iDNA-PKcs, or electroporation only. *n* = 3 CD34^+^ donors from 3 independent experiments. (F) Proportion of GFP^+^ cells in CD34^+^ (left), CD34^+^CD38^–^ (middle), or CD34^+^CD38^−^CD90^+^ (right) HSPC populations at 24 h after editing. *n* = 3 CD34^+^ donors from 3 independent experiments. (G) Representative flow cytometry analysis to quantify HDR. HDR was determined by the proportion of GFP^high^ events, using AAV-only treated cells as a negative control, 5 days after editing. (H) HDR editing efficiency measured by GFP^high^. *n* = 4 CD34^+^ donors from 3 independent experiments. Each colored symbol (C, E, F, and H) represents a unique CD34^+^ donor. AZD7648 (0.1 or 0.3 μM) and rAAV (3.75–7.7 × 10^5^ GC/cell) were delivered at D2 as indicated. (I) Twenty-four hours after the indicated treatment HSPCs were placed into a CFU assay to assess differentiation capacity. After 2 weeks, colonies were enumerated and characterized as BFU-E (burst-forming unit-erythroid), CFU-G/M/GM (colony-forming unit-granulocyte/-macrophage-/-granulocyte-macrophage), or CFU-GEMM (colony-forming unit granulocyte, erythrocyte, macrophage, megakaryocyte) and are represented as a percentage of total CFUs per condition. *n* = 3–4 CD34^+^ donors from 2 independent experiments. (J) Proportion of GFP^+^ colonies from (I) as a percentage of the total colony count per well. CFU plates were imaged using a Cytation 5 and bright-field and fluorescent colonies were counted using ImageJ. For (C), (E), and (F–J), statistical significance was assessed using a linear regression model (see materials and methods). Bars represent mean ± SEM.

After identifying AZD7648 as an enhancer of HDR efficiency with ssODN donor templates in mPB CD34^+^ HSPCs, we next determined the effect of iDNA-PKcs in rAAV-based HDR editing at the *CD40LG* locus. We utilized the RNP targeting *CD40LG* exon 1 (Figure S1; sequence shown schematically in Figure 2A) and an rAAV6 GFP donor template to mediate HDR in mPB HPSCs using the experimental approach shown in Figure 2B. Analysis of cell viability at 24 h post-editing indicated minor toxicity associated with rAAV6 treatment that increased slightly with RNP co-delivery (Figure 2C). Notably, we observed no difference in viability in the presence vs. absence of iDNA-PKcs treatment (Figure 2C). FACS analysis of HSPC surface markers 24 h post-editing showed similar proportions of bulk CD34^+^ HSPCs, CD34^+^CD38^−^ “engraftment enriched” HSPCs,^24^^,^^39^ and CD34^+^CD38^−^CD90^+^ HSC-enriched cells^40^ across treatments (Figures 2D and 2E). Comparison of rAAV6 transduction, as quantified by %GFP^+^ cells in HSPC subsets at day 1 post-editing (as identified in Figure 2E), revealed similar GFP expression levels independent of iDNA-PKcs treatment (Figure 2F). Importantly, HDR rates, based on proportion of GFP^high^ cells at day 5 post-editing, were significantly increased with iDNA-PKcs treatment (Figures 2G and 2H; three experiments, four independent donors). Of note, to optimally account for the variability of starting HDR frequencies across independent experiments and HSPC donors in this and most subsequent experiments, we utilized a linear regression statistical model to determine the significance of the effect of iDNA-PKcs treatment on HDR (see materials and methods).

In addition to characterizing cell viability and HSPC phenotype, cells were removed 24 h after editing and plated in methylcellulose to assess the colony formation potential. To define CFU (colony-forming unit) input cell phenotype, some cells were retained in culture to assess viability, proportion of CD34^+^ and CD34^+^CD38^–^, and GFP (HDR) within CD34^+^ and CD34^+^CD38 populations at day 5 post-editing (Figures S5A–S5E). After 15 days, colonies were enumerated and phenotypically defined. We found that the individual editing components; electroporation (mock-edited), RNP only, AAV only, and iDNA-PKcs impacted total colony formation relative to the untreated control (Figure 2I). The combination of RNP+AAV and RNP+AAV+iDNA-PKcs further limited total colony formation, with a ∼46% and 56% loss in the RNP+AAV and RNP+AAV+iDNA-PKcs groups compared with untreated control, respectively. We observed no statistical difference in colony formation using RNP+AAV vs. RNP+AAV+iDNA-PKcs (*p* = 0.256). We also observed no skewing of colony types between groups (Figure 2I). To specifically test if iDNA-PKcs impacts the colony-forming potential of HDR-edited cells, we counted GFP^+^ colonies to assess %HDR. We found that the increased GFP^+^ population observed at day 5 post-editing in the RNP+AAV+iDNA-PKcs group (Figures S5D and S5E) was maintained at day 15 based on %GFP^+^ colonies (Figure 2J).

Overall, these results demonstrate that iDNA-PKcs treatment significantly increased HDR efficiency in mPB CD34^+^ HSPCs and resulted in minimal additional disturbances in HSPC phenotype or colony formation ability.

### Superior multilineage engraftment of HDR-edited cells with iDNA-PKcs

**Figure 3:**
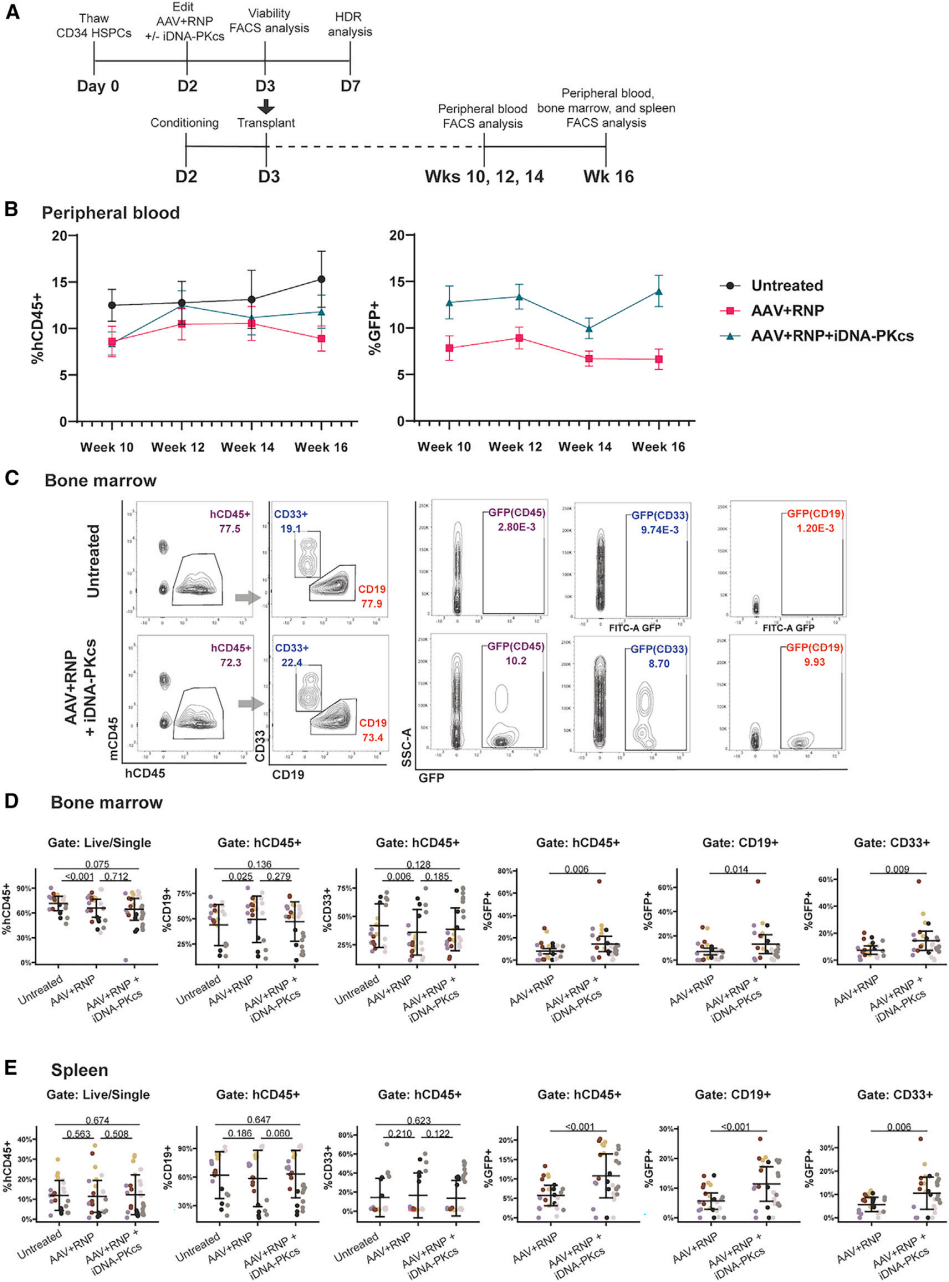
*CD40LG*-edited HSPCs durably engraft with comparable differentiation capacity to untreated HSPCs (A) Schematic for xenotransplantation studies to assess long-term engraftment and differentiation capacity of untreated control, edited, or edited+iDNA-PKcs CD34^+^ HSPCs. Twenty-four hours after editing, HSPCs were transplanted to pre-conditioned NBSGW recipients. Some cells were retained to assess HDR efficiency of input cells. Animals were bled at weeks 10, 12, 14, and 16 to assess engraftment kinetics and frequency of GFP^+^ cells in the periphery. At 16 weeks, BM and spleens are harvested to assess engraftment and %GFP^+^. (B) Mean peripheral blood engraftment (left, % hCD45^+^) and proportion of edited cells (right, %GFP^+^ from hCD45^+^ gate), over time post-transplant. Bars represent ± SEM. (C) Representative analysis of BM from mice transplanted with untreated control or edited+iDNA-PKcs HSPCs. Gating: hCD45 vs. mCD45 gated from live/single cells, and CD19 and CD33 from hCD45^+^. GFP from hCD45^+^, CD33^+^, and CD19^+^ gates is displayed with colors matching parental gate. (D) Analysis of BM 16 weeks after transplant of the indicated treatment groups. Engraftment (%hCD45^+^), proportion of B (CD19^+^) and myeloid lineage cells (CD33^+^), and proportion of edited cells in each subset is depicted; each donor indicated by unique color. Statistical significance assessed by linear regression model. Mean ± SD. (E) Analysis of spleen at 16 weeks. Statistical significance assessed by linear regression model. Mean ± SD. For (B)–(E): *n* = 5 CD34^+^ donors, 6 independent experiments (dark gray and black are same donor repeated). Input %HDR (%GFP^high^ cells assessed at day 5 using an aliquot of the edited cell population cultured *in vitro*) AAV+RNP: 52.2 (gold), 23.4 (light gray), 38.8 (dark gray), and 34.6 (purple). Input %HDR AAV+RNP+iDNA-PKcs: 66.2 (gold), 36.4 (light gray), 47.0 (dark gray), and 43.5 (purple). Input %HDR not recovered due to contamination: black, red. AZD7648 (0.1–0.3 μM) and rAAV6 (3.75–7.7 × 10^5^ GC/cell) were delivered at D2.

To determine the impact of treatment with iDNA-PKcs on the engraftment of HDR-edited mPB HPSCs, we conducted a series of xenotransplantation studies (Figure 3A). Edited (MND.GFP rAAV6+RNP), edited+iDNA-PKcs-treated, or untreated control mPB CD34^+^ HSPCs were transplanted (at 24 h post-editing) into adult NBSGW recipient animals, an immune-deficient strain that supports high levels of human hematopoietic chimerism and multilineage differentiation.^21^^,^^41^

Peripheral blood was analyzed for engraftment (%hCD45^+^) and proportion of edited cells (%GFP^+^; Figure S6A), revealing engraftment across all cohorts (Figure 3B, left). A trend of higher GFP^+^, HDR-edited cells was observed across the duration of the studies with inclusion of iDNA-PKcs (Figure 3B, right). After 16 weeks, bone marrow (BM) and spleens were harvested and analyzed to determine human cell engraftment (%hCD45^+^), differentiation to B cell (%CD19^+^) vs. myeloid lineage (%CD33^+^), and presence of edited cells in each population (Figure 3C). Analysis of recipient BM (Figure 3D) and spleen (Figure 3E) showed engraftment and multilineage differentiation in all animals (left three panels, respectively) and a significantly increased proportion of HDR-edited cells in iDNA-PKcs-treated recipients in both compartments across all lineages (right three panels, respectively).

Together, these observations indicate that mPB CD34^+^ HSPCs edited at the *CD40LG* locus can engraft and differentiate *in vivo* at levels similar to untreated HSPCs. While we observed a limited deficit in BM engraftment with AAV+RNP treatment (mean: 66.5%) relative to untreated control mPB CD34 HSPCs (mean: 72.2%), treatment with iDNA-PKcs had no additional impact (mean: 65.5%). Importantly, these studies demonstrate that the improved HDR efficiency observed *in vitro* with iDNA-PKcs (Figures 2H and 2J) is sustained following engraftment *in vivo* (Figures 3C–3E).

### Sustained, long-term engraftment of *CD40LG* HDR-edited HSPCs

**Figure 4:**
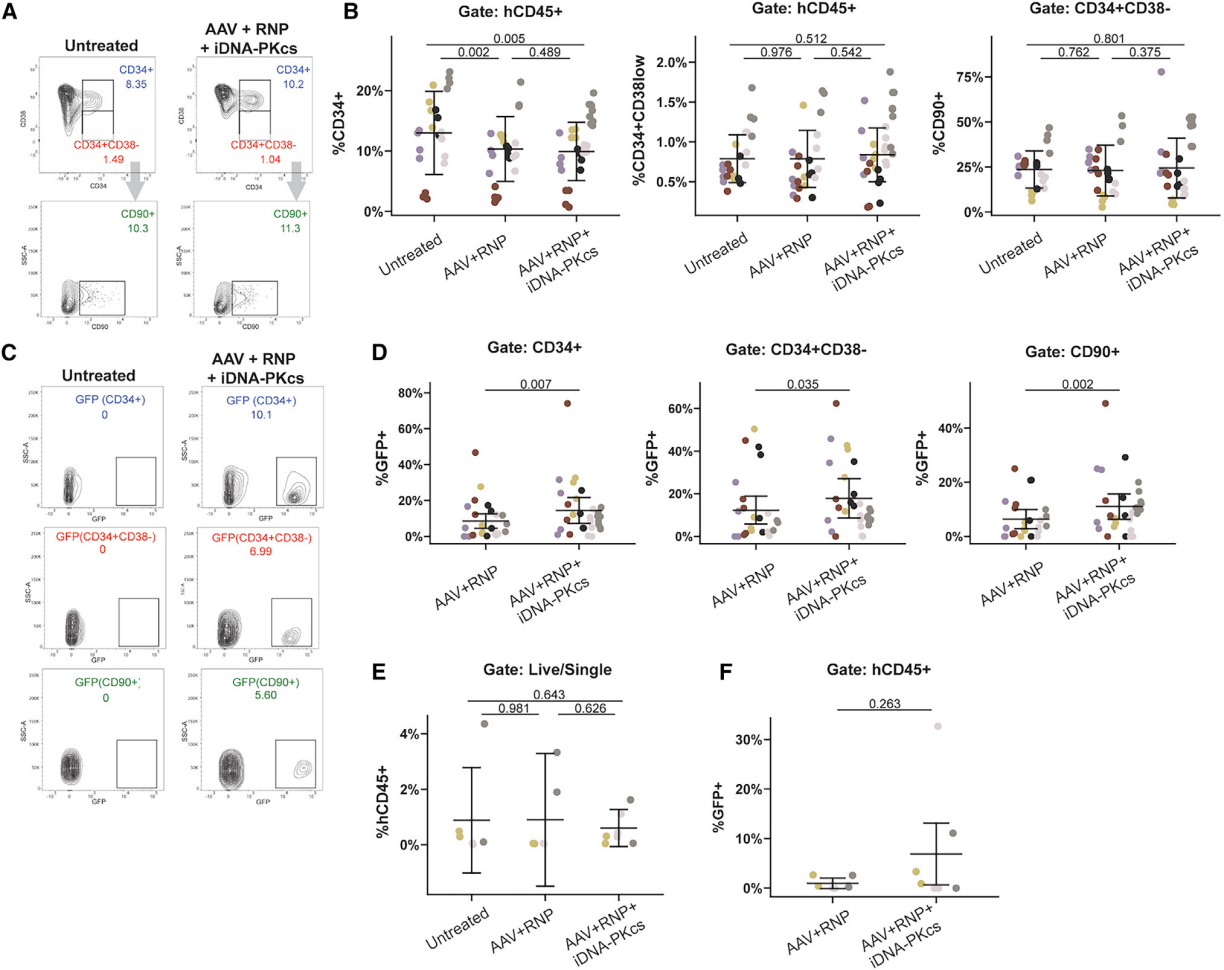
DNA-PK inhibition enhances long-term engraftment of HDR-edited HSPCs (A) Representative flow cytometry analysis of BM HSPC compartment at 16 weeks post-transplant using untreated control or edited+iDNA-PKcs HSPCs. Cells were first gated on live/single/hCD45^+^. CD34 vs. CD38 is plotted to identify bulk CD34^+^ HSPCs and HSC-enriched CD34^+^CD38^–^ HSPCs. CD90 is gated from CD34^+^CD38^–^ to further define a subpopulation of HSC-enriched HSPCs. (B) Proportion of HSPC populations defined in (A) recovered from the BM of transplanted mice. (C) Representative analysis of GFP^+^ cells in HSPC populations defined in (A). (D) Proportion of GFP^+^ HDR-edited HSPCs in populations defined in (A) and (C) recovered from the BM of edited or edited+iDNA-PKcs HSPC-transplanted mice. (E) Equal numbers of BM cells harvested from transplanted animals in (A)–(D) were pooled within treatment groups and transplanted into secondary NSG-SGM3 recipient mice. After 12 weeks, BM was harvested, and engraftment was quantified by %hCD45^+^. (F) Proportion of HDR-edited hCD45^+^ cells recovered from BM of secondary transplant recipients. (A–D) *n* = 5 CD34^+^ donors, 6 independent experiments. (E and F) *n* = 3 CD34^+^ donors, 3 independent experiments. Unique donors are indicated by same color scheme as Figure 3. For (B) and (D), statistical significance was assessed using a linear regression model. Bars represent mean ± SD.

To understand the phenotype and HDR editing frequency of HSPCs engrafted in the BM of recipient mice, we utilized an HSPC-specific immunophenotyping panel (Figure 4A). Consistent with slightly reduced bulk (hCD45^+^) engraftment in AAV-treated conditions, we observed a reduced frequency of total CD34^+^ HSPCs in the AAV+RNP- and AAV+RNP+iDNA-PKcs-recipient animals (Figure 4B). Treatment with iDNA-PKcs had no additional impact on the proportion of CD34^+^ cells compared with HDR editing with AAV+RNP alone. In contrast to bulk CD34^+^ cells, we observed no differences in the proportion of more-primitive, CD34^+^CD38^–^ and CD34^+^CD38^−^CD90^+^ HSPC populations (Figure 4B). Strikingly, analysis of GFP in CD34^+^, CD34^+^CD38^–^, and CD34^+^CD38^−^CD90^+^ populations each showed a significant increase in the proportion of HDR-edited HSPCs with iDNA-PKcs treatment across all donor groups (Figures 4C and 4D).

Next, to stringently characterize the long-term engraftment and self-renewal capacity of HSPCs recovered from NBSGW recipients, we conducted secondary transplant studies utilizing NSG-SGM3 as recipient mice (previously reported to support superior secondary engraftment compared with NSG recipients).^42^ Upon sacrifice at week 16, BM from NBSGW primary recipient animals (shown in Figures 4A–4D) was pooled across groups and transplanted into NSG-SGM3 mice (BM from four primary recipients were pooled and transplanted into two to three secondary recipients). After 12–14 weeks, BM was harvested from secondary recipients and analyzed for human engraftment (Figure 4E) and the proportion of HDR-edited cells (Figure 4F). Comparable levels of engraftment (averaging ∼1%) were observed for all groups (untreated, edited, and edited+iDNA-PKcs). Notably, an average of 0.97% vs. 6.9% HDR-edited cells were recovered from AAV+RNP and AAV+RNP+iDNA-PKcs recipients, respectively. Greater than 1% of HDR-edited cells was present in 2/6 AAV+RNP vs. 3/7 AAV+RNP+iDNA-PKcs recipients. Notably, in two of six AAV+RNP+iDNA-PKcs recipients, but none of the AAV+RNP recipients, the proportion of HDR-edited cells recovered was equal to or greater than the average initial proportion of HDR-edited HSPCs (e.g., within the pooled BM isolated from primary NBSGW recipients). These variable data likely reflect a limited number of HSPCs engrafting in secondary recipients, leading to stochastic variation in HDR. However, recovery of HDR-edited cells from secondary recipients suggests HDR editing in long-lived HSCs. Together, this characterization of primary and secondary xenotransplantation outcomes suggests that iDNA-PKcs supports enhanced HDR editing in primitive, long-term engrafting HSPCs.

### Long-term engraftment and T-lineage differentiation of CD40L cDNA-edited HSPCs

**Figure 5:**
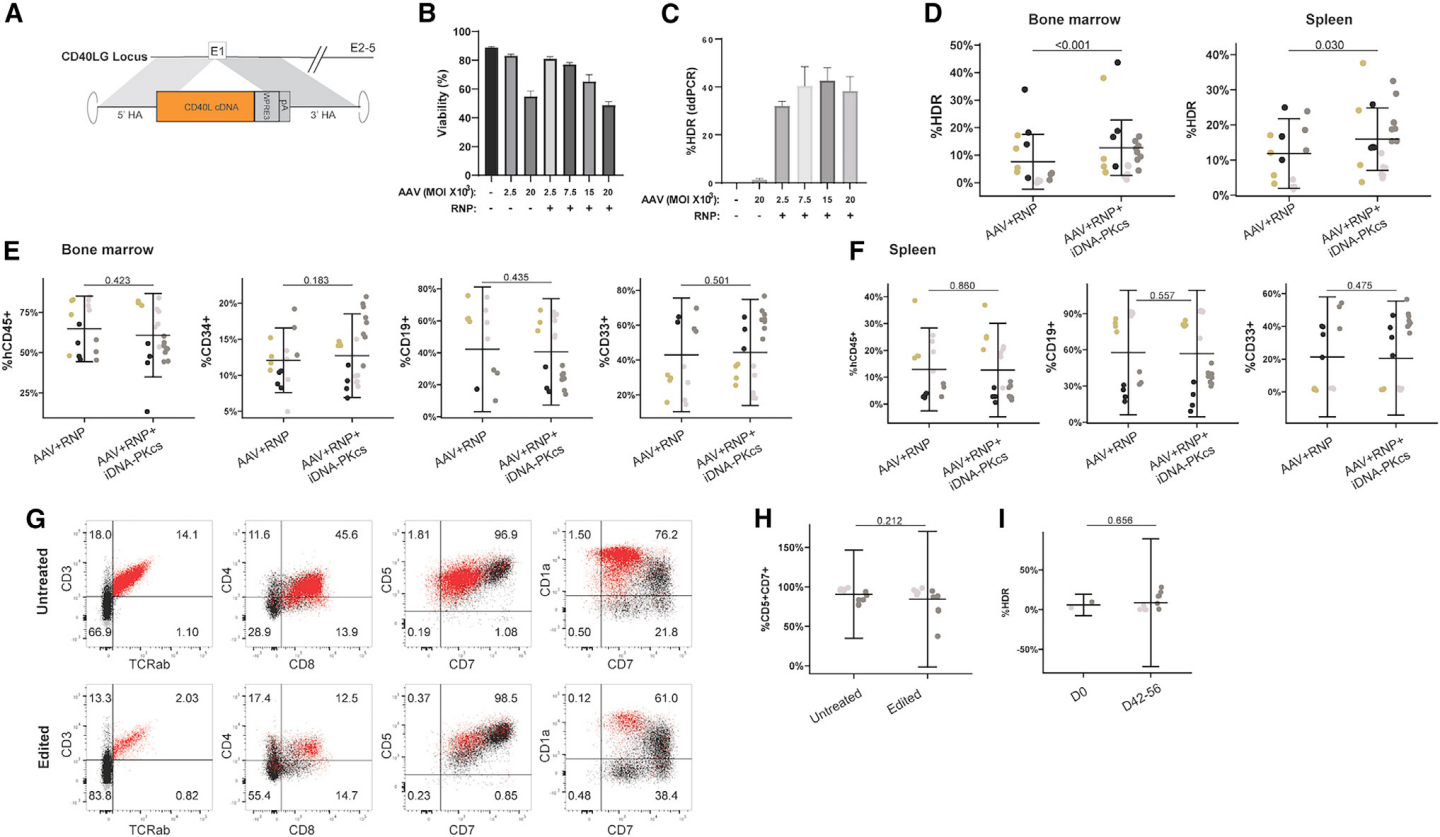
Long-term engraftment and T-lineage differentiation of CD40L cDNA-edited HSPCs (A) Schematic for therapeutic CD40L cDNA HDR donor template. A codon-diverged *CD40LG* coding sequence (cDNA) followed by WPRE3 and SV40USE polyadenylation (pA) elements flanked by 1 kB homology arms is inserted at E1 using the same sgRNA and homology arms described in Figure 2A. Expression of the CD40L cDNA cassette is driven by the endogenous *CD40LG* promoter. (B) Viability measured 48 h after treatment with increasing doses of rAAV6 CD40L cDNA with or without RNP treatment. *n* = 2 CD34^+^ donors, 1 independent experiment. Mean ± SEM (C) HDR measured by ddPCR 5 days after treatment with increasing doses of rAAV6 CD40L cDNA with RNP treatment, or maximal dose or rAAV6 CD40L cDNA without RNP. *n* = 2 CD34^+^ donors, 4 independent experiments. Mean ± SEM. (D) Xenotransplantation studies were conducted as described in Figure 3A utilizing the rAAV6 CD40L cDNA donor cassette ± iDNA-PKcs. After 16 weeks, gDNA was extracted from harvested BM and spleens and the proportion of HDR-edited cells present in the BM (left) and spleen (right) was quantified by ddPCR. Statistical significance assessed by linear regression model. Mean ± SD. (E) Engraftment (%hCD45^+^), and proportion of HSPCs (%CD34^+^), B cells (%CD19^+^), and myeloid cells (%CD33^+^) present in the BM of mice transplanted with edited or edited+iDNA-PKcs HSPCs. Statistical significance assessed by linear regression model. Mean ± SD. (F) Engraftment (%hCD45^+^), and proportion of B cells (%CD19^+^) and myeloid cells (%CD33^+^) present in the spleens of mice transplanted with edited or edited+iDNA-PKcs HSPCs. Statistical significance assessed by linear regression model. Mean ± SD. (G) CD34^+^ cells were isolated from BM harvested from animals in (D) and (E) and differentiated *ex vivo* in artificial thymic organoid (ATO) cultures for 6–8 weeks. In one study, edited samples were pooled between iDNA-PKcs groups (+ and – pooled) due to low CD34^+^ yield (brown color). In the next study, only iDNA-PKcs-treated edited HSPCs were isolated and used to initiate ATO cultures (gray). T-lineage differentiation was characterized by FACS analysis. Cells were first gated by hCD45^+^CD34^–^CD14/CD19/CD56^–^. CD3^+^TCRab^+^ cells are indicated in red and overlaid in FACS plots. (H) Proportion of CD5^+^CD7^+^ T-lineage committed (pre-T-1) cells present at 6–8 weeks after initiation of ATO cultures with CD34^+^ HSPCs isolated from the BM of untreated or edited recipient animals. Statistical significance assessed by linear regression model. Mean ± SD. (I) HDR frequency in pooled input cells (D0) or in individual ATO cultures. Statistical significance assessed by linear regression model. Mean ± SD. (D–F) *n* = 3 CD34^+^ donors (gold, light gray, dark gray/black), 4 independent experiments (dark gray and black are same donor repeated). AZD7648 (0.3 μM) and rAAV (6,300 GC/cell) were delivered at D2. Input HDR (determined by ddPCR at day 5 using an aliquot of the edited cell population cultured *in vitro*) AAV+RNP: 58.9 (gold), 30.6 (light gray), 23.0 (dark gray), and lost from contamination (black). Input HDR AAV+RNP+iDNA-PKcs 64.9 (gold), 44.3 (light gray), 30.0 (dark gray), and lost from contamination (black). (G–I) *n* = 2 CD34^+^ donors, 2 independent experiments.

Moving toward a therapeutic platform for XHIM, we generated an rAAV6 donor template consisting of a codon-diverged *CD40LG* coding sequence (CDS) (identical to coding sequences in Hubbard et al.^11^) and WPRE3 and synthetic poly(A) elements (Figure 5A). Following HDR, expression of the codon-diverged CD40L CDS is designed to be driven by the endogenous *CD40LG* promoter, with the goal of achieving physiological regulation. Using this CD40L “cDNA” donor template, we identified *in vitro* conditions for co-delivery of RNP and rAAV6 donor in mPB CD34^+^ HSPCs that preserved viability (Figure 5B) while maximizing HDR efficiency (Figure 5C).

To determine the long-term engraftment capacity of the mPB CD34^+^ HSPCs edited with this therapeutic donor, we performed xenotransplantation studies in NGSGW recipient mice (Figures 5D–5F). We utilized a similar experimental approach to that detailed in Figure 3A and incorporated HDR editing with or without iDNA-PKcs treatment. We performed four experiments using three CD34^+^ HPSC donors with input HDR rates of 23%–59%. In parallel, we assessed the impact of editing in association with iDNA-PKcs on DNA repair outcomes not resulting in HDR. Consistent with Figure 1C, HDR editing using RNP+AAV in association with iDNA-PKcs resulted in increased rates of MMEJ and a concomitant reduction in NHEJ (Figure S7).

Following adoptive transfer, similar levels of human cell engraftment within the peripheral blood were observed in cDNA-edited vs. cDNA-edited+iDNA-PKcs treated recipients (Figure S8A); levels comparable with engraftment observed with GFP-edited recipients (Figure 3B). Following sacrifice at 16 weeks, HDR in engrafted human cells was assessed using ddPCR (Figure 5D). In comparison with cDNA editing alone, treatment with iDNA-PKcs resulted in significantly higher average levels of long-term engrafted HDR-edited cells in both BM and spleen (BM AAV+RNP: 7.7%; BM AAV+RNP+iDNA-PKcs: 11.9%; SP AAV+RNP: 11.5%; SP AAV+RNP+iDNA-PKcs: 16.2%). For all CD34 donors evaluated, we observed similar human cell engraftment and lineage distribution in BM (Figure 5E) and spleen (Figure 5F).

A constraint of the adult NBSGW humanized model is lack of thymic differentiation of human T cells following transplant of mPB CD34 HSPCs.^21^ Therefore, as an alternative means to assess T cells generated from HDR-edited HPSCs, we utilized an artificial thymic organoid (ATO) system^43^ to assess T-lineage differentiation of BM CD34^+^ HSPCs isolated from long-term engrafted, NBSGW-recipient mice. Human CD34^+^ cells were enriched from BM pools from recipient mice (two independent transplant experiments) and used to initiate ATO cultures. Following 6–8 weeks in culture, T cell differentiation was characterized by immunophenotyping (Figures 5G and 5H). T-lineage cells across a range of differentiation stages, including abundant CD5^+^CD7^+^ pre-T1 cells as well as DP and limited numbers of SP T cells, were present in cultures initiated using either untreated or CD40L cDNA-edited HSPCs (Figure 5G, upper vs. lower panels). To determine whether HDR-edited HSPCs contributed to the generation of T-lineage cells, %HDR was quantified by ddPCR in ATOs (after 6–8 weeks) and compared with %HDR of input CD34^+^ HSPCs isolated from long-term engrafted, cDNA-edited, recipient animal BM (Figure 5I). While variable across the studies, the proportion of HDR-edited T cells was similar to the proportion of edited cells within the input HSPC population. These findings indicate that long-term engrafted HSPCs recovered from xenotransplantation studies retain T-lineage differentiation capacity and that HDR editing at the *CD40LG* locus does not impair their differentiation capability.

### T cells derived *in vivo* from *CD40LG*-edited HSPCs exhibit regulated CD40L expression

**Figure 6:**
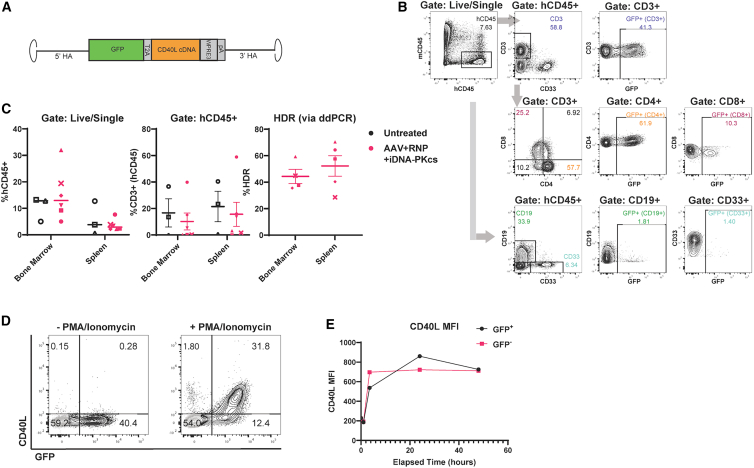
*Ex vivo* stimulation of T cells derived from *in vivo* differentiation of CD40L-edited HSPCs in NSG-SGM3 mice (A) Schematic of rAAV6 CD40L.GFP donor template consisting of a codon-diverged CD40L cDNA 2A-linked to a GFP reporter followed by WPRE3 and SV40 poly(A) sequences with flanking by 1 kB homology arms. Expression of the knockin cassette is driven by the endogenous *CD40LG* promoter. (B) Representative flow cytometry analysis of splenic cells in an NSG-SGM3-recipient mouse at 16 weeks post-transplant of CD40L.GFP+iDNA-PKcs-edited HSPCs. (C) Human cell engraftment (%hCD45^+^; left panel) and T cells (%CD3^+^; middle panel) quantified by flow, and HDR quantified by ddPCR (right panel) in BM and spleen. Symbols indicate individual animals. Bars represent mean ± SEM. (D) Flow cytometry plots showing CD40L and GFP co-expression 3.5 h after PMA and ionomycin stimulation of splenocytes harvested from an CD40L.GFP+iDNA-PKcs-edited HSPC-recipient animal. (E) CD40L MFI in GFP^+^ and GFP^−^ quadrants from (D) measured at 0, 1, 3.5, 24, and 48 h after PMA and ionomycin stimulation. (B–E) Representative data from 1 of 2 experiments. AZD7648 (0.3 μM) and rAAV (6690 GC/cell) were delivered at day 2.

Characterization of CD40L cDNA expression is critical to infer the functionality and safety of CD4 T cells expressing a candidate therapeutic HDR cassette. Moving toward an HSPC editing therapy and to allow for direct tracking of HDR-edited, CD40L-expressing T cells, we generated an alternative AAV donor cassette containing a GFP.T2A.CD40L cDNA expression cassette (Figure 6A). This *cis*-linked GFP donor design was utilized to permit flow-based tracking of naive T cells derived from HDR-edited HPSCs *in vivo*. mPB CD34^+^ HSPCs were edited in association with iDNA-PKcs treatment using this new donor as detailed in Figure 3A. In comparison with the NSG or NBSGW strains, the NSG-SGM3 mouse strain has been reported previously to support more efficient T cell differentiation from engrafted human HPSCs.^42^ Therefore, to facilitate generation of T cells *in vivo* from HDR-edited adult mPB HPSCs, we performed transplant studies using this strain. Busulfan-conditioned, adult NSG-SGM3-recipient mice were transplanted with unedited or edited HPSCs and recipient mice were evaluated at 16 weeks post-transplant. Similar levels of human chimerism were observed in recipients transplanted with unedited vs. edited HSPCs (Figures 6B and 6C). As expected, based on previous studies, overall human cell chimerism was lower in this strain (Figures 6B and 6C) compared with NBSGW recipients shown in Figures 3, 4, and 5. Variable proportions of CD3^+^ T cells were present in both the BM and spleen across both cohorts. Notably, following long-term engraftment, HDR rates in both BM (44.3%) and spleen (52.3%) were similar to the HDR level of input HSPCs (63.4%) (Figure 6C), demonstrating sustained engraftment of CD40L HDR-edited, iDNA-PKcs-treated, adult mPB HPSCs in this alternative model.

Consistent with intracellular, endogenously regulated, CD40L expression that occurs in naive human T cells, flow cytometry of a CD40L cDNA.GFP+iDNA-PKcs-treated recipient revealed T cell-specific, baseline *cis*-linked GFP expression driven off the integrated HDR cassette (Figure 6B). Next, to determine whether HDR-edited T cells exhibit activation-dependent surface CD40L expression, we used a modified activation protocol.^11^ We characterized transgenic CD40L cDNA expression kinetics by stimulating with PMA and ionomycin to activate surface CD40L expression and tracked expression based on co-expression of GFP and CD40L (CD154) surface staining. As T cell numbers were limited, we activated total spleen cells and stained for surface CD40L expression. Activation led to a CD40L^+^GFP^+^ double-positive population. Comparing CD40L expression in GFP^+^ vs. GFP^–^ populations revealed a similar level of CD40L surface expression (based upon CD154 MFI) and comparable expression kinetics consistent with endogenous CD40L expression in response to T cell activation (Figures 6D and 6E). Together, these data demonstrate sustained engraftment of *CD40LG* HDR-edited mPB HPSCs, differentiation of edited HPSCs into naive T cells *in vivo*, and endogenously regulated CD40L surface expression following *ex vivo* activation of T cells derived from HDR-edited mPB HPSCs.

## Discussion

HDR-based gene editing to precisely insert a therapeutic expression cassette in HSPCs has emerged as a potential future therapeutic strategy for monogenic blood and immune disorders.^19^^,^^20^^,^^44^ Here, utilizing a high specificity, small-molecule inhibitor of DNA-PKcs, AZD7648, in association with co-delivery of CRISPR-SpCas9 RNP and an rAAV6 homology template, we established an efficient HDR-based HSPC gene editing approach to treat XHIM syndrome. Using clinically relevant, mPB CD34^+^ HSPCs, we achieve precise insertion of a therapeutic CD40L cDNA cassette at the *CD40LG* locus and long-term engraftment of CD40L-edited HSPCs *in vivo*, at predicted therapeutically beneficial levels. Importantly, this approach permits T cell differentiation *in vivo* and *in vitro* and leads to endogenous promoter-regulated CD40L expression kinetics.

Previously, we reported efficient HDR editing at the *CD40LG* locus in primary T cells using TALEN and rAAV6.^11^ By targeting *CD40LG* exon 1, the upstream promoter and intronic regulatory elements are conserved, enabling regulated expression of the rAAV6-delivered DNA donor cassette. Here, we designed an sgRNA proximal to the previously reported TALEN site and compared *CD40LG* editing efficiency and viability in mPB CD34^+^ HSPCs. At matched rAAV6 doses, we found superior HDR editing efficiency and higher cell viability with SpCas9 nuclease. Next, we optimized HSPC culturing parameters through addition of small molecules SR1 and UM171^32^^,^^33^ that increased the proportion of primitive CD34^+^CD38^−^CD90^+^CD133^+^ HSPCs. Optimization of HSPC culturing and nuclease choice yielded roughly 40% HDR *in vitro* using a therapeutic CD40L cDNA cassette. In previous work,^12^ a similar strategy was used to introduce a CD40L cDNA leading to efficient HDR in HSPCs and XHIM patient-derived T cells *in vitro*. However, transplant of HDR-edited HSPCs yielded limited long-term persistence of HDR-edited cells *in vivo*; comparable with levels reported here in the absence of iDNA-PKcs. The authors speculated that long-term engraftment may be driven by a quiescent fraction of HSPCs resistant to HDR. An alternative HDR strategy has also been used previously to target intron 1 of the *CD40LG* gene for HDR editing^13^; an approach designed to avoid unregulated expression from off-target integration but not capable of addressing mutations within exon 1. Using cord blood CD34^+^ HSPCs, the authors reported efficient HDR and engraftment of HDR-edited cells at higher levels when editing was performed in association with inhibition of p53 via GSE56 mRNA^45^^,^^46^; observations that highlight the potential clinical benefit of transiently targeting the DNA damage response (DDR).^47^ Combinatorial inhibition of DNA-PKcs and p53 (via GSE56 mRNA or via alternative approaches) in mPB CD34^+^ HSPCs could be considered in future studies.

While efficient HDR has now been reported for many genetic loci *in vitro*, reduced *in vivo* engraftment of HDR-edited HSPCs remains a major hurdle to clinical translation.^15^^,^^16^^,^^17^^,^^47^^,^^48^ This challenge is especially important using clinically relevant HSPCs including mPB CD34^+^ cells. Engraftment-enriched CD34^+^CD38^–^ HSPCs are more quiescent relative to bulk CD34^+^ HSPCs and skew toward NHEJ repair, rather than HDR, in the context of gene editing.^24^ Small-molecule inhibitors of DNA-PKcs, an enzyme critical in facilitating NHEJ repair, have been used to enhance HDR in cell lines and primary cell populations.^27^^,^^29^^,^^30^^,^^31^^,^^49^ In this study, we demonstrate that AZD7648, a highly selective inhibitor of DNA-PKcs,^28^ potently inhibits NHEJ allowing for enhanced HDR rates in HSPCs with both ssODN and rAAV6 DNA donor templates. We achieved up to 60% HDR at the *CD40LG* locus with rAAV6 DNA donors with sustained benefit in CFU assays. Most notably, using a series of xenotransplantation studies, iDNA-PKcs co-delivery improved the *in vivo* recovery of long-term repopulating, HDR-edited, HSPCs. Analysis of BM from recipient NBSGW animals at 16 weeks showed ∼70% higher levels of HDR-edited CD34^+^CD38^−^CD90^+^ HSPCs following editing in the presence of iDNA-PKcs. Consistent with our findings, Selvaraj et al. identified AZD7648 to be superior to alternative DNA-PKcs inhibitors in enhancing the HDR:indel ratio in cord blood CD34^+^ HSPCs *in vitro* using an rAAV6/RNP editing strategy, findings that correlated with an increased proportion of HDR-edited CFU colonies.^29^ While the authors did not perform xenotransplantation studies, they speculated that AZD7648 might increase engraftment of gene-targeted HSPCs. This speculation is validated by our *in vivo* findings.

Inhibition of 53BP1 in CD34^+^ HSPCs has been evaluated as an alternative approach to limit NHEJ and promote HDR.^50^^,^^51^ Transient delivery of i53 mRNA^52^ led to an ∼50% increase in HDR efficiency at the *CYBB* locus, and combination of i53 and GSE56 mRNAs resulted in 76% greater HDR efficiency at the *MAGT1* locus and an ∼50% increase in HDR-edited cells recovered in NSG-SGM3 recipients. In contrast to AZD7648 small-molecule delivery, mRNA delivery requires generation of high-purity, non-immunostimulatory products to limit innate RNA sensing. Interestingly, across multiple groups, maximal mean HDR efficiency with rAAV6 templates reach ∼60%–70% in CD34^+^ HSPCs. Of note, combinatorial inhibition of DNA-PKcs and PolΘ mediated further HDR enhancement by simultaneously targeting NHEJ and Θ-mediated end joining^30^; however, this approach has not been assessed for *in vivo* efficacy using HPSCs. Inhibition of Θ-mediated end joining has also been reported to reduce the frequency of large deletions occurring after DSB generation.^53^ Overall, targeted inhibition of NHEJ provides a key tool for enhancing HSPC HDR editing, a conclusion strongly supported by our combined *in vivo* findings.

While targeting DNA repair mechanisms can partially address the deficit in engrafted HDR-edited CD34^+^ HSPCs, other mechanisms likely also limit the clinical translation of rAAV6-based HSPC editing. In this study, we observe modest evidence for rAAV6-associated toxicity *in vivo* in comparison with mice transplanted with control HSPCs. rAAV6 exposure triggers a p53-dependent DDR in HSPCs that can be partially mitigated via transient p53 inhibition.^45^^,^^46^^,^^50^^,^^54^ While rAAV likely directly mediates a transient DDR, AAV ITR fragments trapped at on- and off-target sites DSBs may also impact the p53-dependent DDR.^54^ Interestingly, DNA-PK has been implicated in a STING-independent DNA-sensing pathway.^55^ The proportional increase in HDR observed *in vivo* suggests that AZD7648, in addition to promoting HDR, may play a role in limiting toxicity in HSPCs sensitive to rAAV6-assocaited toxicities. Future work is required to determine whether a combination of AZD7648 and p53 inhibition, or targeting other innate signaling pathways, can additionally mitigate impacts of rAAV6 exposure. Potential candidate small-molecule inhibitors may include those tested in primary T cells in association with a double-stranded DNA donor template.^56^

While rAAV6 remains the primary method for delivering large DNA donors for HDR editing in HSPCs, HDR mediated by alternative DNA templates might be similarly potentiated with AZD7648. Consistent with this concept, we observed a ∼50% increase in HDR rates in edited mPB CD34^+^ HSPCs using AZD7648 in association with ssODNs. While relatively inefficient in CD34^+^ HSPCs, single- or double-stranded DNA donors may also exhibit improved efficiency in combination with AZD7648. Consistent with this concept, combined use of inhibitors of DNA-PKcs, HDAC1/2, and CDC7 facilitated knockin of non-viral, DNA donors in primary T cells.^57^ Furthermore, IDLV-based editing can achieve HDR efficiencies comparable with rAAV6^54^ and might be similarly facilitated with AZD7648 delivery.

Achieving physiological rescue of CD40L expression is critical for successful XHIM therapy, as unregulated CD40L expression results in lymphoproliferative disease.^6^ The CD40L cDNA *cis*-linked GFP cassette utilized in our study mediated T cell-specific expression and activation-dependent CD40L surface expression by T cells differentiated from CD40L-edited mPB HSPCs *in vivo*. This design was similar to our previous work in primary T cells.^11^ However, here we altered the donor cassette by placing the GFP and codon-diverged CD40L cDNA bicistronic elements downstream of the DNA cut site and utilized a truncated WPRE3 element. In T cells, derived *in vivo* from mPB CD34^+^ HSPC transplant recipients, *cis*-linked GFP was specifically expressed in unstimulated CD3^+^ cells, consistent with endogenous, lineage-specific, *CD40LG* promoter activity leading to intracellular loading of CD40L-containing vesicles. Furthermore, we demonstrate physiological, T cell activation dependent, CD40L surface expression. In a similar study,^13^ CD40L surface expression was shown following stimulation of CD4^+^ T cells recovered from mice transplanted using HDR-edited cord blood CD34^+^ HSPCs. These combined findings support the safety and function of our therapeutic CD40L cDNA donor, confirming T lymphocyte differentiation *in vivo* and physiological *ex vivo* activation following editing at the *CD40LG* locus and long-term engraftment of mPB CD34^+^ HSPCs.

Importantly, we focused our studies on HDR editing and transplantation of clinically relevant, mPB CD34^+^ HSPCs into adult recipient animals to most accurately model future therapeutic strategies. One challenge with existing xenotransplantation models (particularly in using intravenous delivery of adult HSPCs in adult recipient mice) is the limited ability to achieve sustained engraftment of primitive HSPCs in parallel with efficient T cell differentiation. While robust T cell development is observed following intrahepatic transplant in neonatal recipients,^50^^,^^58^ T cell development following transplantation of mPB CD34^+^ HSPCs into adult immune-deficient animals is more limited.^21^ Conversely, while alternative models, including NSG-SGM3 mice, permit more efficient T cell development, HSC engraftment progressively declines over time.^42^ As an overall approach to assess both CD34^+^ HSPC engraftment and T cell differentiation, we used a combinational approach of NBSGW and NSG-SGM3 models, primary and secondary transplants, and *in vitro* ATO studies using CD34^+^ HSPCs recovered post-transplant. In the NSG-SGM3 model, we observed T-lineage-specific GFP expression consistent with endogenously regulated expression. Notably, we also recovered higher levels of HDR-edited cells in the NSG-SGM3 model; levels nearly equivalent to input HDR rates. This observation is consistent with other work^50^^,^^59^ and likely reflects expression of human cytokines that promote lineage-committed, HDR-edited human progenitors, rather than more primitive CD34^+^CD38^–^ HSPCs. To stringently characterize editing in long-term engrafted HSPCs, we performed secondary transplant studies, transplanting BM from primary NBSGW recipients into secondary recipient NSG-SGM3 animals. This approach was based upon previous findings^42^ indicating superior secondary engraftment in NSG-SGM3 mice. GFP^+^ edited cells were recovered in secondary recipients showing HDR editing in long-term engrafted HSPCs. Despite the limited human chimerism and small number of secondary recipients studied, our findings suggest that iDNA-PKcs may facilitate increased long-term engraftment of HDR-edited HSPCs. Moving forward, it will also be important to assess the clonality of HSPCs engrafted *in vivo*^46^ to further assess the safety of proposed editing strategies for XHIM.

In summary, we demonstrate an SpCas9-based HDR editing strategy to deliver functional CD40L cDNA to the *CD40LG* locus. Inclusion of the specific DNA-PKcs inhibitor, AZD7648, led to improved levels of long-term engrafted, HDR-edited, mPB HSPCs approaching levels predicted to provide clinical benefit in HIGM^14^ and generated T cells with endogenous promoter-regulated CD40L expression. Prior to therapeutic application, additional considerations to reduce toxicity and enhance long-term engraftment of HDR-edited HSPCs will be necessary. Given the ease of AZD7648 delivery, it should be feasible to apply additional steps to mitigate the DDR or innate immune response and/or further modulate DNA repair pathways to achieve meaningful advances toward clinical application. Taken together, our findings suggest that AZD7648 co-delivery may improve therapeutic editing across a range of nuclease and DNA donor platforms in multiple hematopoietic diseases.

## Materials and methods

### Nuclease reagents

*CD40LG* TALENs were identical to those described previously in Hubbard et al.,^11^ except that they were cloned into a pEVL backbone rather than a pUC57 backbone, which was linearized using *Bsa*I. TALEN mRNAs were transcribed from the linearized plasmids and capped (5′ 7-methylguanylate cap with cap-1 structure) *in vitro* using the T7 mScript mRNA Production System (CellScript Madison, WI) following the manufacturer’s instructions and as described previously.^60^^,^^61^ Final purification was performed using NucleoSpin RNA clean-up (Machery Nagel, Germany). TALENs were electroporated at 50 μg/mL for each TALEN. SpCas9RNPs targeting *CD40LG* or *B2M* were generated using Alt-R V3 SpCas9 (Integrated DNA Technologies, Coralville, IA) and the following sgRNAs: *CD40LG* 5′-AAAGUUGAAAUGGUAUCUUC’-3'; *B2M* 5ʹ-CGAUAAGCGUCAGAGCGCCG-3ʹ. In Figures 1, S4, and S5, SpCas9 RNPs were made by incubating the SpCas9 protein with sgRNA synthesized by IDT at 2:1 sgRNA:SpCas9 ratio for 10–15 min at 37°C. SpCas9 RNP was electroporated at a concentration of 150 μg/mL. For all other experiments, SpCas9 protein was incubated with sgRNAs from Synthego (Redwood City, CA) at 1:1.2 M ratio for 15 min at room temperature, and the resulting SpCas9 RNP was electroporated at a concentration of 80 μg/mL reaction volume. For Figure 1, the following ssODN donor template for *B2M* (IDT) was included in the RNP complexing reaction: 5′-AAAGAGCGGA AGAGAAACCC TCCCCCAACC TCGGCTCGAG CGCTCTGACG CTTATCGACG CCCTAAACTT TGTCCCGACC-3ʹ.

### rAAV6 design and production

Plasmids were adapted from those described previously in Hubbard et al.^11^

To generate the pAAV.MND.GFP.WPRE construct, the MND-modified retroviral promoter was inserted into the previously described pAAVCD40LG[GFP.WPRE] plasmid. The full CD40L.cDNA.WPRE3.SV40PolyA was synthesized (GeneArt) and cloned into a pAAV backbone with 1 kB *CD40LG* homology arms by infusion cloning (Clontech). The pAAV.CD40LG[GFP.2A.CD40L cDNA.WPRE3]^11^ was cloned into a pAAV backbone with 1 kB *CD40LG* homology arms. AAV6 vector stocks were produced and titered as described previously.^62^

### HSPC culture and editing

mPB CD34^+^ HSPCs (CD34-purified peripheral blood stem cells apheresed from G-CSF-mobilized adult male donors) were purchased from Fred Hutchinson Cancer Research Center (Seattle, WA) or Charles River Laboratories (Wilmington, MA). For most experiments, cells were thawed and cultured at a concentration of 0.25 × 10^6^ cells/mL in HSPC growth medium: serum-free expansion medium II (SFEMII) (STEMCELL Technologies, Vancouver, CA) supplemented with TPO, SCF, FLT3-L, IL-6 (100 ng/mL each, Preprotech, Waltham, MA) and 1 μM SR1 (STEMCELL Technologies) and 35 nM UM171 (ApexBio, Houston, TX). However, experiments in Figures S1 and S2 used a cell density of 1 × 10^6^ cells/mL and serum-free stem cell growth medium (CellGenix, Sartorius, Germany) in place of SFEMII; experiments in Figures S1 and S4 did not include SR1 or UM171 in the growth medium.

For the experiments shown in Figures S1 and S2, cells were cultured for 48 h at 37°C, resuspended in Neon Buffer T (Thermo Fisher Scientific), then electroporated with either TALEN mRNA or SpCas9 RNP (1,400 V, 20 ms, 1 pulse) using a Neon Transfection System (Thermo Fisher Scientific). Immediately after electroporation, cells were plated at a concentration of 5 × 10^5^ cells/mL in fresh HSPC growth medium with rAAV6 added 1–5 × 10^5^ GC (viral genome copies)/cell. 24 h after editing, AAV-containing medium was removed and replaced with fresh medium.

In all other experiments, cells were cultured for 48 h at 37°C, resuspended in P3 Buffer (Lonza, Switzerland) then electroporated with the 4D-Nucleofector system (Lonza) using CM149 or DZ100 electroporation protocols. Cells were rested for 5–10 min post-electroporation, then plated at a concentration of 1 × 10^6^ cells/mL. rAAV6 was added at 1.5% of the final culture volume (unless otherwise noted) and MOI (genome copies [GC]/cell) was determined based on viral titer. AZD7648 in DMSO (Selleck Chemicals, Houston, TX) was diluted in SFEMII prior to addition to rAAV6-containing medium at a concentration of 0.3 μM unless otherwise noted. Twenty-four hours after editing, AAV- or AZD7648-containing medium was removed and replaced with fresh medium and analyzed by flow cytometry to assess viability, %GFP^+^, and HSPC phenotype using the following antibodies: CD34, CD38, CD90, CD133. For Figure 1, viability was determined using BD Via-Probe Red Nucleic Acid Stain (BD Sciences). Otherwise, viability was determined by FSC vs. SSC. Five days after editing, cells were analyzed by flow cytometry to assess viability and %GFP^high^ to determine HDR.

### CFU assays

Twenty-four hours after editing, cells were counted and processed for CFU assays. Viability and cell number were determined on the CytoflexS (Beckman Coulter) with DAPI used as a viability dye and the recording set to volume to determine cells/mL. Thereafter, the viable cells/mL concentration was used to plate an equal number of cells per condition to establish the impact of each individual gene editing component and the combinations thereof on colony formation.

Viable cells from each condition were seeded at 250 cells per well in 6-well SmartDish (STEMCELL Technologies) in MethoCult H4034 Optimum (STEMCELL Technologies) according to the manufacturer’s instructions and incubated at 37°C for 14 days. Plates were imaged using a STEMVision (STEMCELL Technologies) instrument and dark and bright-field images were processed for CFU determination using the STEMVision Analyser software using the 14-day EPO huPB program. Colony assignments (BFU-E, CFU-G/GM/GM, and CFU-GEMM) by the software were further interrogated by eye using the ColonyMarker software (STEMCELL Technologies). Colony counts are depicted as stacked histograms, with each stack representing the different colony assignments. The cumulative stack represents the total colony count per condition for each well evaluated. To determine impact on colony type, each colony type was normalized to total colony counts and represented as a percentage of the total for each treatment group.

GFP-expressing colonies were acquired using a Cytation 5 imager (Biotek, Agilent Technologies, Santa Clara, CA). Images were processed using the Gen 5 Secure Image Prime software (BioTek, Agilent Technologies). The following settings were used for image acquisition: temperature set to 37°C; bright-field conditions set to illumination 10, integration time 5, and gain 3.3; and GFP conditions set to illumination 10, integration time 5, and gain 22.8. Images were acquired in montage mode with 11 × 11 (columns × rows) images to cover the entire six wells. Tile overlap was set to auto for stitching. After acquisition, images were processed for stitching using the linear blend method with a reduction of the total image size to 20% of the original image. GFP^+^ colonies were counted using ImageJ multipoint tool (ImageJ 1.53t, National Institutes of Health). The %GFP^+^ was calculated as the numbers of GFP^+^ colonies divided by total colonies (as assessed on the STEMVision) × 100.

### Xenotransplantation

NBSGW and NSG-SGM3 mice were purchased from Jackson Laboratory (Bar Harbor, ME) and maintained in a pathogen-free facility at Seattle Children’s Research Institute. All animal studies were approved by Seattle Children’s Research Institute’s Institutional Animal Care and Use Committee (IACUC) and were performed in accordance with the National Institutes of Health Guide for the Care and Use of Laboratory Animals. For primary transplants in NBSGW mice, 6- to 10-week-old female mice were treated with 12. 5 mg/kg busulfan 24 h prior to cell transplant. Twenty-four hours after editing, 1.5–2 × 10^6^ cells were delivered retro-orbitally (equal doses across treatment groups). Nonmanipulated cells were transferred as control. Peripheral blood was collected 10, 12, 14, and 16 weeks post-transplant and analyzed by flow cytometry. After 16 weeks post-transplant, the mice were euthanized. Leukocytes collected from the BM and spleen were used for immunophenotyping and to extract genomic DNA (gDNA) for molecular analyses. To assess engraftment and differentiation, cells were analyzed by flow cytometry after labeling with antibodies against human antigens CD45, CD33, and CD19, as well as murine CD45 (antibody reagents are further described in Table S4; all antibodies recognize the human antigen unless specified). LT-HSCs were phenotypically detected by staining with CD34, CD38, and CD90 antibodies.

For primary transplant into the NSG-SGM3 strain, 8- to 10-week-old female mice were treated with 25 mg/kg busulfan 24 h prior to cell transplant. Twenty-four hours after editing, 2 × 10^6^ cells were delivered retro-orbitally. Mice were euthanized at 14 weeks post-transplant and assessed as above; splenocytes were also stimulated in culture with PMA and ionomycin (below).

In some cases, BM cells from the NBSGW primary transplanted mice were serially passaged into NSG-SGM3 mice (secondary transplant recipients). Here, 6- to 8-week-old female NSG-SGM3 mice were treated with 25 mg/kg busulfan 24 h prior to cell transplant. On the day of secondary transplant, BM from NBSGW primary xenotransplant recipients was extracted and pooled within treatment groups. BM cells (2 × 10^6^) were transplanted retro-orbitally to recipient NSG-SGM3 mice. After 12–14 weeks, BM was harvested and immunophenotyping performed to assess the presence of the xenograft and HDR-edited cells.

### ATO cultures

ATO cultures were performed following the protocol described by Montel-Hagen et al.^43^ Cultures were initiated with 5,000 CD34^+^ HSPCs isolated with a CD34 microbead kit (Miltenyi Biotec, Germany) from pooled BM of recipient NBSGW mice. Input cells were analyzed by FACS to confirm purity and ddPCR was performed on gDNA extracted to quantify input HDR rate. Cultures were maintained for 6–8 weeks. After 6–8 weeks, cultures were harvested for gDNA extraction and ddPCR analysis to quantify HDR or FACS analysis to assess differentiation using the following antibodies: hCD45, CD34, CD14, CD56, CD19, CD1a, CD7, CD3, TCRαß, CD4, CD8.

### PMA ionomycin stimulation of NSG-SGM3 splenocytes

Splenocytes isolated from primary-transplanted NSG-SGM3 mice were cultured at 1–2 × 10^6^ cells/mL and stimulated with 50 ng/mL PMA and 1 μg/mL ionomycin (Sigma-Aldrich, St Louis, MO). After stimulation, cells were washed in PBS, then stained with the following antibodies: CD45, CD33, CD19, CD4, CD8, CD154 (CD40L) and Live/Dead-Near IR (ThermoFisher Scientific).

### Quantification of HDR by ddPCR

Genomic DNA was isolated from *in vitro* cultured HSPCs or BM and splenic leukocytes using a DNeasy Blood and Tissue kit (QIAGEN, Germany). To quantify HDR editing, “in-out” ddPCR was performed with the forward primer binding within the knockin cassette and reverse primer binding outside the region of homology. A control amplicon of similar size (1.3 kB) was generated for the *ActB* gene (Table S3). Probes for both amplicons were labeled with FAM and amplified in separate ddPCR reactions in duplicate. The PCR reaction included 900 nM each primer (IDT), 250 nM probe (IDT), 50 ng gDNA, and ddPCR Supermix for probes without dUTP (Bio-Rad). Droplets were created using a QX200 Droplet Generator (Bio-Rad). Droplets were analyzed using the QX200 Droplet Digital PCR System (Bio-Rad) and analyzed using Quantasoft (Bio-Rad). As *CD40LG* is on the X chromosome and all CD34 donors were male, editing rates were calculated as a ratio of copies/μL from *CD40LG*/*ActB* multiplied by 2.

### Sequencing analysis of *B2M* locus

gDNA was extracted from CD34^+^ HSPCs 48 h after editing using QuickExtract DNA Extraction Solution (Biosearch Technologies, UK). The concentration of the resulting gDNA sample was measured using the Qubit 3.0 Fluorometer (Invitrogen, MA). PCR amplification of the edited region was performed using primers flanking the *B2M* target region (Table S3). After amplification, the PCR product was visualized by electrophoresis on the E-Gel Power Snap System (Invitrogen) in accordance with the manufacturer’s instructions to confirm the presence of the expected ∼500 bp bands. gDNA samples with detectable bands were subjected to Sanger sequencing (Genewiz, Azenta Life Sciences). Sequencing was performed using the instructions for "Difficult Templates" (Genewiz, Azenta, Life Sciences). Deconvolution of the trace data was analyzed for indel frequency and or insertional frequency using ICE analysis (Synthego). Editing efficiency rates (i.e., percentage of the pool with non-WT sequence) were determined by comparing the edited trace with a control (*B2M*) trace. Indels were counted and graphed as WT (0), NHEJ (+/−1, +/−2), or MMEJ (−3 ≤; ≥3). Analysis of indels and integration by ICE was also used to infer the dominant DNA repair pathway. HDR was inferred from template integration, MMEJ dominance inferred from large deletions (i.e., ≥3 bp deletion), and NHEJ dominance inferred from small indels (i.e., ≤3 bp indel).

### Sequencing analysis of the *CD40LG* locus

gDNA was extracted from *in vitro* cultured CD34^+^ HSPCs 5 days after HDR editing using DNeasy Blood and Tissue kit (QIAGEN). PCR amplification of the edited region was performed using primers flanking the *CD40LG* target region (Table S3). After amplification, PCR products were visualized by electrophoresis by Gel Doc XR+ (Bio-Rad) in accordance with the manufacturer’s instructions to confirm the presence of the expected ∼300 bp (non-HDR repair) and ∼1.8 kB (HDR) bands. The ∼300 bp band was extracted with QIAquick Gel Extraction kit (QIAGEN) and subjected to Sanger sequencing (Genewiz, Azenta Life Sciences). Deconvolution of the trace data was analyzed for indel frequency using ICE analysis (Synthego). Indels were counted and graphed as WT (0), NHEJ (+/−1, +/−2), or MMEJ (−3 ≤; ≥3).

### Flow cytometry

Flow cytometry analysis was done with an LSR II (BD Biosciences) or MACSquant Analyzer (Miltenyi Biotec) and analyzed with FlowJo V.10.8.1 (BD Biosciences). Antibody information is included in Table S4.

### CAST-seq off-target analysis

CAST-seq was performed with the *CD40LG* sgRNA in mPB CD34^+^ HSPCs of a single donor in four replicates (duplicates of SpCas9 RNP preparation and electroporation, duplicates of each edited cell population). mPB CD34^+^ HSPCs were thawed and cultured for 3 days prior to editing. HSPCs were electroporated with SpCas9 RNPs using a 4D-Nucleofector (Lonza). With each SpCas9 RNP duplicate, 2 × 10^5^ cells were electroporated. Four days post-electroporation, each cell population was split 1:2, resulting in duplicates of each duplicate nucleofection (total of four replicates). Five days post-electroporation, genomic DNA was extracted. T7E1 cleavage assays as well as ICE indel analyses were performed with two replicates to confirm efficient on-target editing. With all four replicates, CAST-seq library preparations for next-generation sequencing were performed as described in Turchiano et al.,^34^ and data were analyzed using an improved bioinformatics pipeline.^63^

### Statistical analysis

GraphPad Prism v. 9.1.1 (GraphPad Software, CA) was used to plot graphs and for statistical analyses. For HDR, NHEJ, and AAV dose curves, a four-parameter logistic non-linear regression model was used to determine the IC_50_ (NHEJ) and EC_50_ (HDR and AAV) values.

Relationships between treatment (untreated, AAV, AAV+RNP, AAV+RNP+iDNA-PKcs) and experimental readouts were assessed using a linear regression model in Figures 2C, 2E, 2F, 2H, 2J, 3D, 3E, 4B, 4D, 4E, 5D–5F, 5H, 5I, and S5A–S5E. Readouts (continuous variables) were considered as dependent variables, whereas treatment was considered as an independent variable (categorical variable). Since the results are clustered by donor and study, the model used incorporated a random intercept to account for variations in baseline level of the readouts between the different clusters formed by donors and studies (i.e., all donor and study combinations). In addition, we incorporated a robust estimate of the variance to account for potential between-treatment heteroscedasticity. For Figures 2J and 4F, a fixed intercept model was used because it was not possible to fit random intercept models for the corresponding readouts. For Figures 2I and S5F, the cell type (BFU-E, CFU-G/M/GM, or CFU-GEMM) was added to the model as a fixed effect in interaction with the treatment effect.

## Data and code availability

Data supporting the findings of the present study are available upon request from the corresponding author.

## Supplemental information

Document S1. Figures S1–S8 and Tables S1–S4

Document S2. Article plus supplemental information
