# Supplementary material for: DNA-PK inhibition enhances gene editing efficiency in HSPCs for CRISPR-based treatment of X-linked hyper IgM syndrome
Source: Mol Ther Methods Clin Dev. 2024 Jul 27;32(3):101297. doi: 10.1016/j.omtm.2024.101297 (PMC11863497; doi:10.1016/j.omtm.2024.101297)
Supplement: Document S1. Figures S1–S8 and Tables S1–S4 [file mmc1.pdf]

OMTM, Volume *32*

Supplemental information

DNA-PK inhibition enhances gene editing efficiency in HSPCs for CRISPR-based treatment of X-linked hyper IgM syndrome

Cole M. Pugliano, Mason Berger, Roslyn M. Ray, Kai Sapkos, Betty Wu, Aidan Laird, Yidian Ye, Daniel Thomson, M. Quinn DeGottardi, Iram F. Khan, Kristina Tatiossian, Brodie A. Miles, Florian Aeschimann, Jerome Pasquier, Mihee M. Kim, and David J. Rawlings

Supplemental Materials

Supplemental Figures

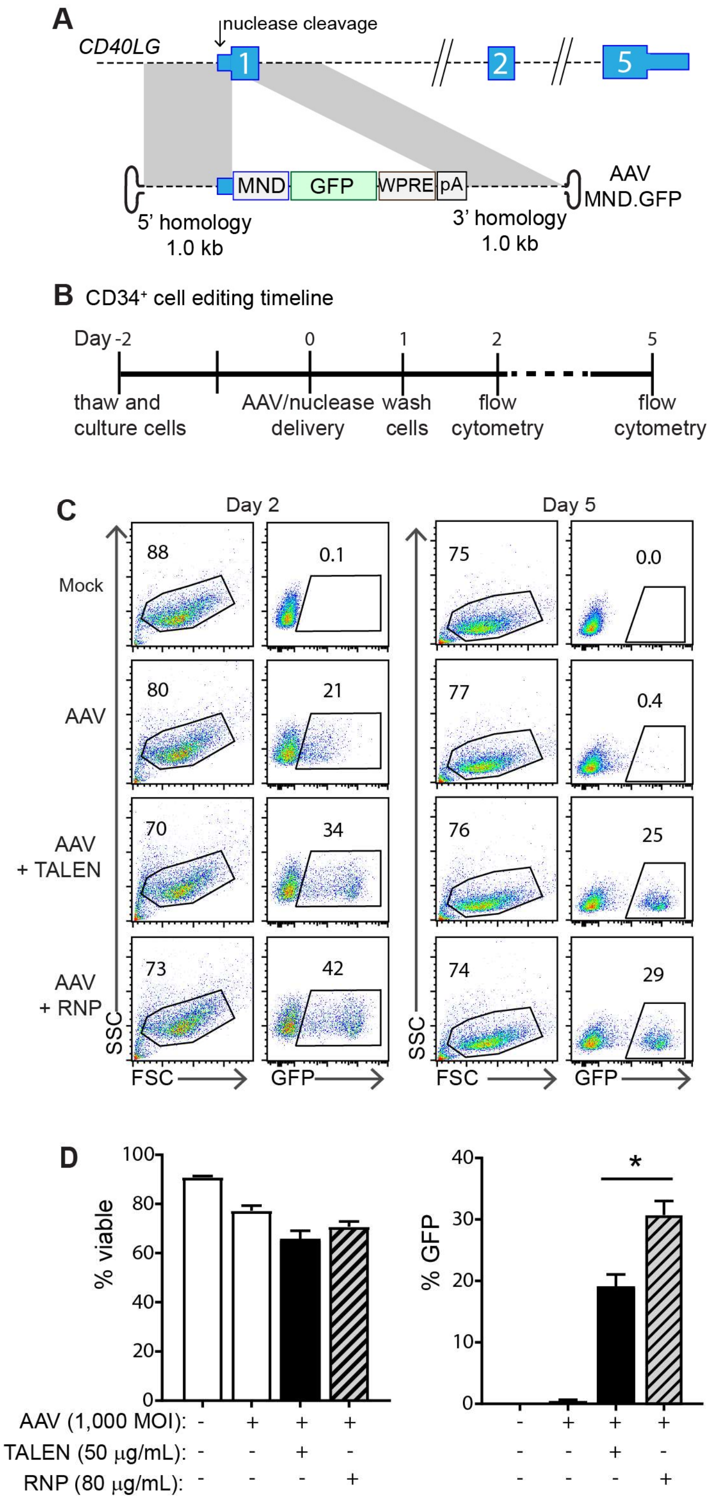

Figure S1. Establishment of CRISPR Cas9-based HDR editing at CD40L locus in CD34+ HSPCs.

(A) Human *CD40LG* locus showing nuclease recognition site relative to exons (blue boxes) and translated coding sequence (taller blue boxes); below is AAV donor template with MND.GFP reporter; grey shading shows location of *CD40LG* homology. (B) Experimental timeline: day 0 is designated as editing (electroporation with Cas9 RNP or TALEN mRNA) followed by culturing in media containing AAV donor template. (C) Representative flow cytometry plots for mock, AAV only (MOI=1000) and AAV (MOI=1000) and TALEN (50 µg/ml) or RNP (80 µg/ml) conditions showing gating for viability (SSC vs. FSC) and GFP expression within the live cell gate (SSC vs. GFP) on the indicated day post-editing. (D) Mean ± SEM cell viability (*left*) and percentage of GFP^+ edited cells (*right*) as determined by flow cytometry 2 and 5 days post-editing, respectively (N=9 replicates with 4 unique donors).
P value was determined using unpaired t-test. *$p \leq 0.05$.

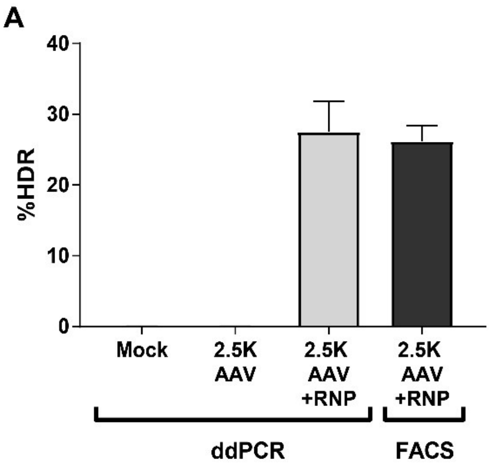

Figure S2. Confirmation of On-Target Integration of MND.GFP Reporter.
(A) Average editing rates using MND.GFP reporter template in $CD34^+$ donors as determined by ddPCR vs. flow cytometry for GFP. Data are presented as mean ± SEM (N=5 replicates with 2 unique donors).

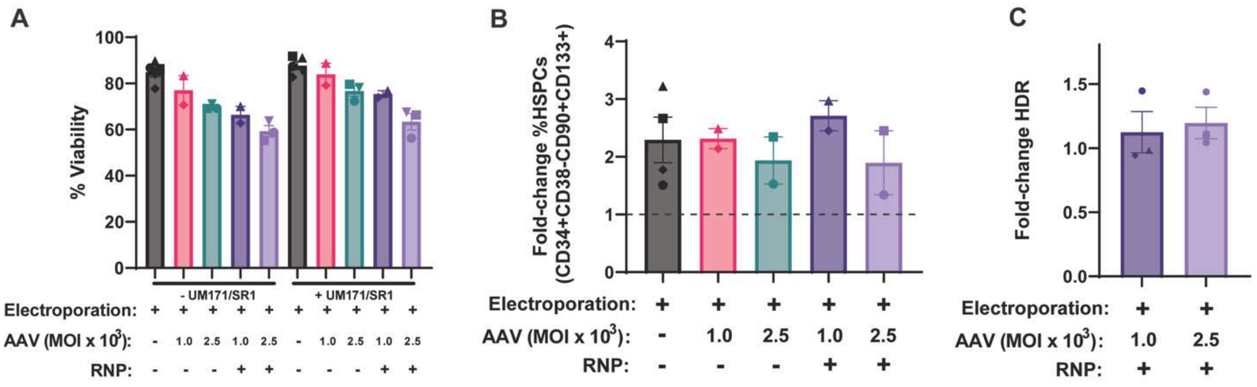

Figure S3. Incorporation of UM171 and SR1 within the *in vitro* CD34+ HSPC culturing protocol. (A) Viability (measured by FSC-A vs. SSC-A) 48h after mock-treatment (electroporation), AAV-treatment, or AAV+RNP editing of HSPCs cultured in the presence or absence of UM171 (35nM) and SR1 (1uM). (B) Fold-change in the percentage of HSC-enriched CD34+CD38-CD90+CD133+ HSPCs in cultures 48h after editing reagent treatment. For each editing condition, the frequency of HSC-enriched HSPCs in the UM171/SR1 group is normalized to the same treatment without UM171/SR1. (C) Fold-change in HDR efficiency with UM171 and SR1 treatment 5 days after editing (a normalized to the same treatment without UM171/SR1). (A-C) n=2-4 CD34+ donors, 2-3 independent experiments Bars represent mean +/- SEM.

A

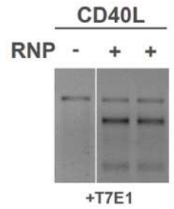

Target/sample	T7E1 Cleavage [%]	ICE Indels [%]
CD40L-T-1-1	81	92
CD40L-T-1-2	78	94

B

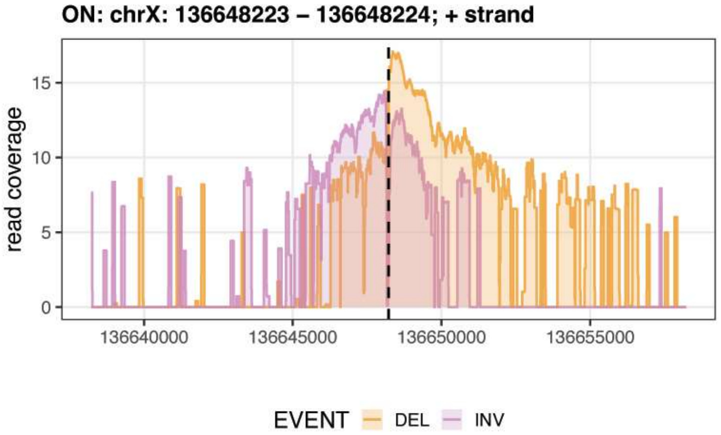

C

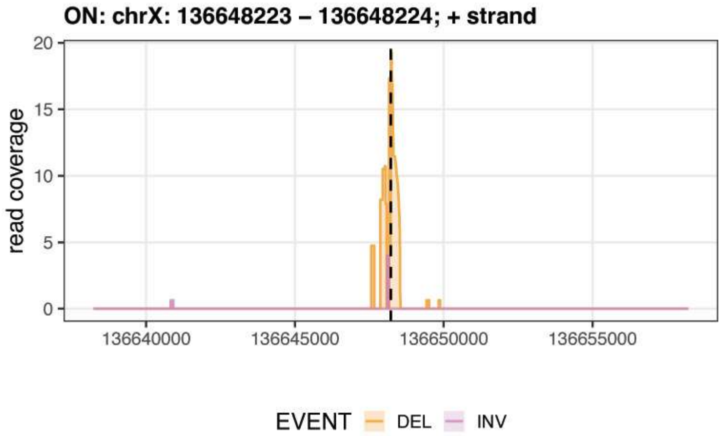

D

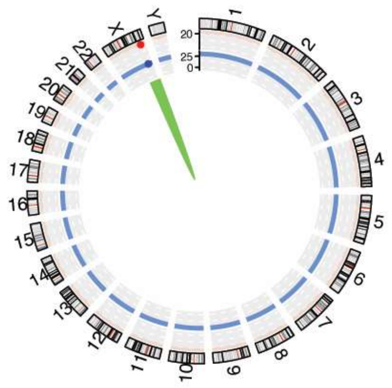

E

chromosome	start	end	read	hits	NB.OVL	NB.SIGNIF	aln.sum	score	SYMBOL
chrX	136635647	136671020	12320605	38653	4	4	aaagttgaaatggtatcttc**TGg**	20,5	LINC00892
chr11	104512736	104513246	3385	8	1	1	aaagttgaaatAAt-tctCc**ATg**	10,5	LINC02552
chr14	86800615	86801125	465	5	2	1	aaaAttg++aaatgCta-cttc**AAg**	10,5	LINC01148
chr6	108880332	108880832	3943	4	2	1	aaTgttgaTGtggtGGcttc**AGg**	10,5	ARMC2
chr12	9260454	9260954	3880	1	1	1	Gaagtt+++gaaat+g+gtatcttc**AGg**	10,5	LINC00987

querry: aaagttgaaatggtatcttcNRG Capital letters=mismatch; - =deletion; + =insertion; bold=PAM

Figure S4. CAST-seq analysis of *CD40LG*-targeting sgRNA.
(A) T7E1 cleavage assay and ICE indel analysis to verify on-target editing in two of the four replicates (labeled CD40L-T-1-1 and CD40L-T-1-2 in the table) of CD34+ mPB HSPCs subjected to CAST-seq analysis (see Materials and Methods). (B) Large deletions (DEL) and inversions (INV) induced by Cas9 cleavage at the on-target site in a window including 10kb upstream and downstream of the cleavage site. Depicted is the sum of all four edited samples. (C) Large deletions (DEL) and inversions (INV) detected in untreated control samples at the on-target site in a window including 10kb upstream and downstream of the cleavage site. Depicted is the sum of all four samples. (D) Circos plot visualizing the specificity of the *CD40LG*-targeting sgRNA, with the on-target site cluster shown in green. Red and blue layers represent the alignment and homology scores, respectively. No OMTs were detected with application of the

following criteria: 1) OMTs must have a read:hit ratio of >10, to remove non-specifically assigned reads/sites. 2) OMTs have to be identified in 3 samples (NB.OVL) and be significant in 2 samples (NB.SIGNIF). (E) Off-target-mediated translocations (OMTs) detected by CAST-Seq in table format. Results are shown for OMTs with scores > 10, i.e. the four identified low-probability off-target sites (score = 10.5), as well as for the on-target site (highlighted in green). Reads: absolute number of NGS reads that passed the CAST-Seq filter. Hits: the number of individual translocation events detected. Those are defined as fusions of the same two chromosomal regions but with a unique molecular signature, i.e. with a distinct translocation fusion point (due to differences in the DNA repair outcome) or with a distinct linker ligation point. NB.OVL: number of replicates with the detected OMT. NB.SIGNIF: number of replicates with the detected OMT with significant *p* value. Aln.sum: Alignment to 23 bp sgRNA target sequence (red capital letter = mismatch; "-" = deletion; "+" = insertion; bold = PAM). Score: score of the alignment to the sgRNA target site (min score = 8, max score = 20.5). SYMBOL: gene closest to the fusion point.

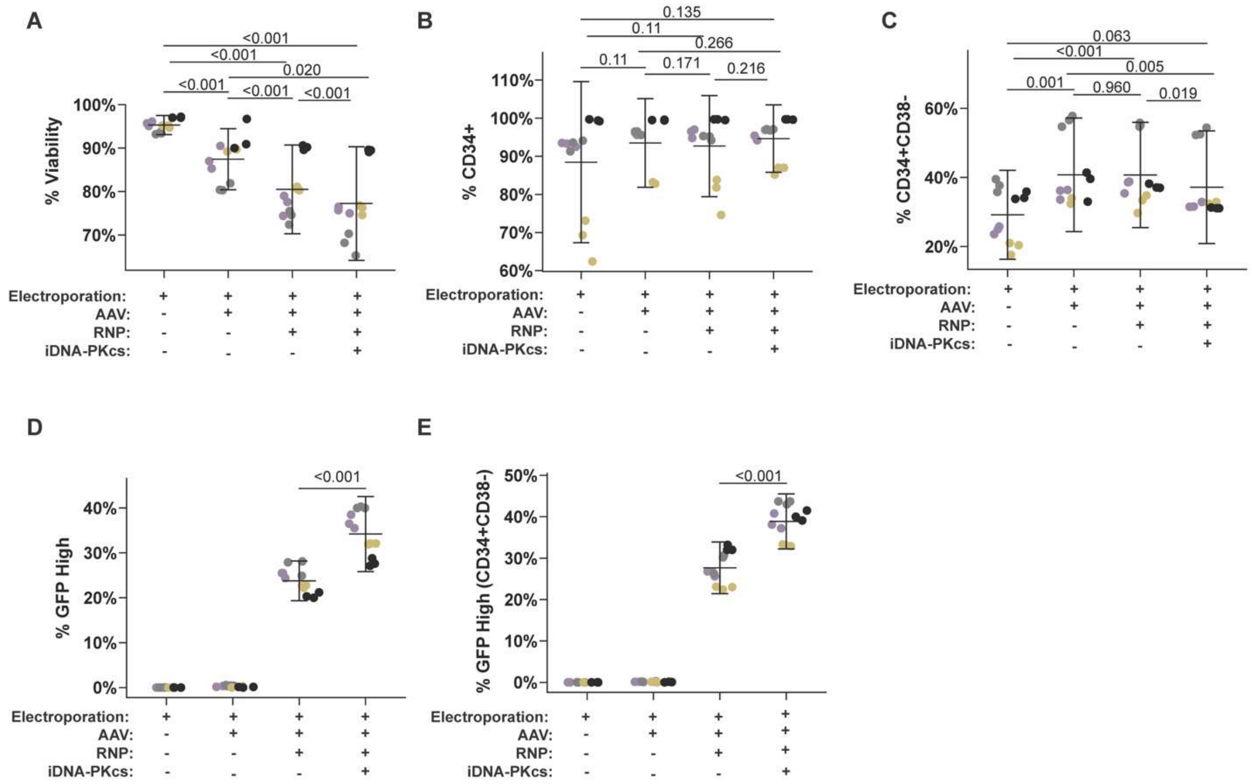

Figure S5. **Characterization of cell populations used to establish CFU assays after editing.** (A) Viability measured by FACS (FSC-A vs. SSC-A) 5 days after electroporation alone, electroporation in association with AAV delivery, HDR editing, or HDR editing in combination with iDNA-PKcs. (B) Proportion of $CD34^+$ and (C) $CD34^+CD38^-$ HSPCs as percentage of total cells 5 days after editing. (D) Proportion of GFP+ cells in $CD34^+$ cells and (E) Proportion of GFP+ cells in $CD34^+CD38^-$ cells in HSPC populations 5 days after editing. (A-E) n=4 $CD34^+$ donors from 2 independent experiments. Statistical significance was assessed using a linear regression model (See Materials and Methods). Bars represent mean +/- SEM.

A

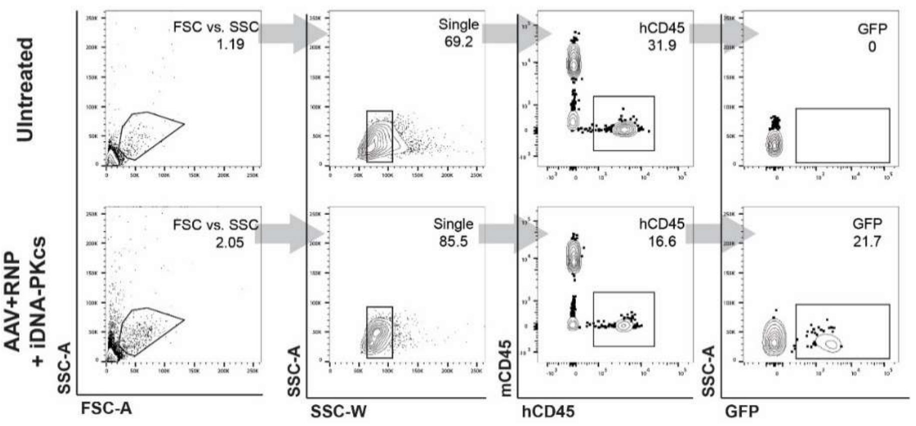

Figure S6. Peripheral blood FACS analysis.
(A) Gating scheme for peripheral blood FACS analysis at 10,12,14, and 16 weeks post-transplant.

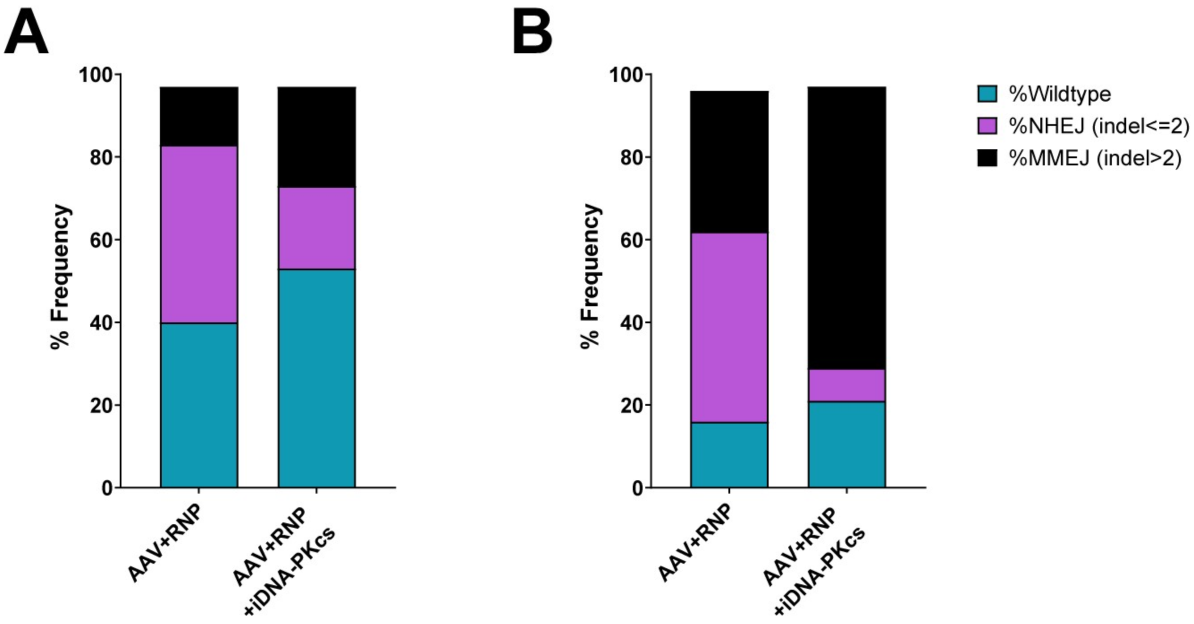

Figure S7. **Indel profiling of rAAV6+RNP-based editing outcomes with or without DNA-PKcs addition.**
Indel frequencies following HDR editing using rAAV6+RNP vs. rAAV6+RNP with AZD7648 were evaluated using ICE analysis (see Methods). Indels ≤ ±2 bp were categorized as NHEJ, while indels ≥ ±3 bp were defined as MMEJ. Histograms represent the percentages of WT, NHEJ, and MMEJ events for each treatment group. (A-B) are representative of n = 2 CD34+ donors with analysis performed in 2 independent experiments using HDR edited cells from 2 of the 3 donors shown in Figure 5. A portion of each HDR edited cell population was retained and cultured in vitro for 5 days prior to extraction of gDNA (A, corresponds to donor depicted in gold color and B, donor depicted in dark grey, in Fig. 5).

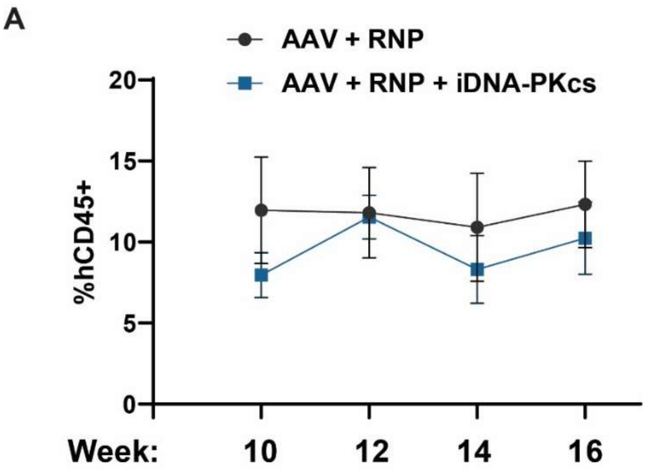

Figure S8. Peripheral blood engraftment of CD40L cDNA-edited HSPCs.
(A) Percentage of hCD45+ cells in peripheral blood of mice transplanted with CD40L cDNA-edited or CD40L cDNA + iDNA-PKcs-edited HSPCs.
n=3 CD34+ donors, 4 independent experiments
Points represent mean +/- SEM

Supplemental Tables

Table S1. NHEJ Non-linear 4-PL fit data

	NHEJ donor 1	NHEJ donor 2	NHEJ donor 3	NHEJ donor 4	NHEJ donor 5	NHEJ donor 6
Top	58.22	60.54	21.51	56.74	52.21	28.6
Bottom	8.01	14.47	-0.1803	10.46	14.63	-0.4719
Log IC_{50}	-6.861	-6.779	-6.848	-6.564	-6.924	-6.815
HillSlope	-3.657	-2.389	-3.265	-2.53	-5.807	-2.053
IC_{50}	1.377E-07	1.664E-07	1.419E-07	2.728E-07	1.191E-07	1.531E-07
R^2	0.9584	0.9866	0.9245	0.9688	0.8953	0.9676

Table S2. HDR Non-linear 4-PL fit data

	HDR donor 1	HDR donor 2	HDR donor 3	HDR donor 4	HDR donor 5	HDR donor 6
Top	84.04	77.27	49.43	74.42	87.84	48.98
Bottom	40.96	47.81	29.37	30.11	44.64	22.96
Log IC_{50}	-6.178	-7.064	-6.929	-6.949	-7.31	-7.027
HillSlope	0.4719	2.037	1.667	1.683	1.074	1.787
EC_{50}	6.632E-07	8.626E-08	1.178E-07	1.125E-07	4.897E-08	9.408E-08
R^2	0.9136	0.9284	0.7146	0.8284	0.6624	0.9455

Table S3. Oligonucleotide sequences (5' to 3').

**For ddPCR detection of *CD40LG* MND.GFP HDR**	
**Forward primer**	ACCTGTCAGCTCCTTTCC
**Reverse primer**	GGTCCAGATCCTAAGAGAGG
**Probe**	TCAATCCAGCGGACCTTCCT
**For ddPCR detection of CD40L cDNA and GFP.T2A HDR**	
**Forward primer**	AGTGTCTTCGTCAACGTGACAG
**Reverse primer**	TCCCTGATAAAGTGCAATCATCC
**Probe**	TCCTGGTTAGTTCTTGCCAC
**For ddPCR detection of Beta Actin (*ActB*)**	
**Forward primer**	ACTCTGCAGGTTCTATTTGC
**Reverse primer**	AATGATCTGAGGAGGGAAGG
**Probe**	ATCAAGGTGGGTGTCTTTCC
**For amplifying and sequencing *B2M***	
***B2M* forward primer**	GCGTGAGTCTCTCCTACCCTC
***B2M* reverse primer**	CCTAGACGAAGTCCACAGCTC
***B2M* sequencing primer**	CTCTGGTCCTTCCTCTCCCG
**For indel profiling of rAAV6+RNP based HDR at *CD40LG***	
***CD40LG* forward primer**	CGTAACGTTTTTGCTGGGAGAG
***CD40LG* reverse primer**	GCAAAAAGTGCTGACCCAATCA

Table S4. Antibody Reagents

Antibody Target*	Fluorophore	Vendor	Catalog #
CD1a	PerCP Cy5.5	BioLegend	300130
CD3	BV786	BioLegend	344842
CD4	BV605	BioLegend	300556
CD7	FITC	BioLegend	343104
CD8	Alexa700	BioLegend	344724
CD14	PECy7	Thermo Fisher Scientific	25-0149-42
CD19	PECy7	BioLegend	302215
CD33	PE	BD Biosciences	555450
CD34	APC Cy7	BioLegend	343514
CD38	PerCPCy5.5	BD Biosciences	551400
CD45	eFluor 450	Thermo Fisher Scientific	48045941
Murine CD45	APC	Thermo Fisher Scientific	17-0451-82
CD56	PECy7	BioLegend	318318
CD90	APC	BD Biosciences	561971
CD90	PECy7	BD Biosciences	561558
CD133	PE	Miltenyi Biotec	130-080-801
CD154 (CD40L)	PE	eBioscience	12-1548-42
TCRαß	PE	BioLegend	306708

*all antibodies target human proteins unless specified
